# Action of multiple intra-QTL genes concerted around a co-localized transcription factor underpins a large effect QTL

**DOI:** 10.1038/srep15183

**Published:** 2015-10-28

**Authors:** Shalabh Dixit, Akshaya Kumar Biswal, Aye Min, Amelia Henry, Rowena H. Oane, Manish L. Raorane, Toshisangba Longkumer, Isaiah M. Pabuayon, Sumanth K. Mutte, Adithi R. Vardarajan, Berta Miro, Ganesan Govindan, Blesilda Albano-Enriquez, Mandy Pueffeld, Nese Sreenivasulu, Inez Slamet-Loedin, Kalaipandian Sundarvelpandian, Yuan-Ching Tsai, Saurabh Raghuvanshi, Yue-Ie C. Hsing, Arvind Kumar, Ajay Kohli

**Affiliations:** 1 Plant Breeding, Genetics and Biotechnology Division, International Rice Research Institute, DAPO 7777, Metro Manila-1226, Philippines; 2 Leibniz Institute of Plant Genetics and Crop Plant Research (IPK), Corrensstrasse 03, 06466 Gatersleben, Germany; 3 Institute of Plant and Microbial Biology, Academia Sinica, 128 Sec. 2, Academia Road, Nankang, Taipei 11529, Taiwan; 4 Department of Plant Molecular Biology, University of Delhi South Campus, New Delhi, 110021, India

## Abstract

Sub-QTLs and multiple intra-QTL genes are hypothesized to underpin large-effect QTLs. Known QTLs over gene families, biosynthetic pathways or certain traits represent functional gene-clusters of genes of the same gene ontology (GO). Gene-clusters containing genes of different GO have not been elaborated, except *in silico* as coexpressed genes within QTLs. Here we demonstrate the requirement of multiple intra-QTL genes for the full impact of QTL *qDTY*_*12.1*_ on rice yield under drought. Multiple evidences are presented for the need of the transcription factor ‘no apical meristem’ (*OsNAM*_*12.1*_) and its co-localized target genes of separate GO categories for *qDTY*_*12.1*_ function, raising a regulon-like model of genetic architecture. The molecular underpinnings of *qDTY*_*12.1*_ support its effectiveness in further improving a drought tolerant genotype and for its validity in multiple genotypes/ecosystems/environments. Resolving the combinatorial value of *OsNAM*_*12.1*_ with individual intra-QTL genes notwithstanding, identification and analyses of *qDTY*_*12.1*_has fast-tracked rice improvement towards food security.

Drought recurrently causes natural plant population and agricultural crop failure. Drought has had long-term, irrevocable adverse affects on two billion and killed 11 million people in the last century, more than any other natural hazard^1^. Rice (*Oryza sativa*) is particularly sensitive to water deficit and exhibits significant yield losses even under mild drought^2^. Rice and its cultivation is the main source of food and livelihood for the millions of poor on marginal rainfed lands prone to drought, increasingly so under the exacerbated droughts predicted by climate-change scenarios^3^.

Conventional, marker-assisted, and transgenic breeding approaches exist for generating drought tolerant rice. Although precision genome engineering is continually evolving, inhibitory costs and intractable philosophies weigh down transgenic product development. Conventional breeding is temporally demanding. With temporal and technical advantages, marker-based QTL mapping and field tests combined with candidate gene cloning seems more practicable for crop improvement. However, limited examples exist of cloning the cardinal gene underpinning plant QTLs^4^. For drought tolerance, QTLs *Pro-W2*^5^ and *Dro1*^6^ have been cloned from Arabidopsis and rice, respectively. Nonetheless, validity of *Dro1* or other QTLs is uncertain in multiple genotypes/environments/ecosystems. Indeed, genotypes with the same functional allele of *Dro1* exhibited different root growth angles^7^.

Various rice breeding lines, morpho-physiological traits, QTLs, and genes are known for drought tolerance^8^,^9^,^10^. Despite such promising reports and significant investment over the decades, high yielding rice varieties that are more drought tolerant than the available landraces are rare. This is mainly because the identified factors were mostly valid at the vegetative stage, with no effect on yield under stress. Also, different rice varieties inhabit different eco-geographies. For example, genomic distinctions exist between the *O. sativa* sub-classes *indica*, *temperate japonica*, *tropical japonica* and *aus*^11^, which have regional and topographical niches in the four ecosystems of irrigated, rainfed lowland, rainfed upland and flood prone rice cultivation areas. The inter-specific NERICA rice arising from the *O. sativa* x *O. glaberrima* cross is largely grown in Africa. Therefore, most factors identified for drought tolerance may not have consistent effects in different environments and in different genetic backgrounds. Minor-effect QTLs may also thus be specific to environmental and genetic niches. Such QTLs are generally underpinned by a single gene such as the *Dro1*, which are increasingly seen to be part of a set of minor QTLs^7^. Genetic, genomic, and/or molecular characterization of the interactions among such minor QTLs is challenging due to the intricate and inter-dependent network of effects at the transcript, protein, and metabolite levels, which confound the results.

Large-effect QTLs comprise multiple small-effect QTLs, especially for complex traits^12^ and large-effect QTLs for traits under abiotic selection are more stable^13^. Attempts at identifying large-effect QTLs with broad utility in crop improvement are a relatively recent phenomenon and even more recent is the limited success in such attempts. We reported a large-effect QTL on rice chromosome 12 for yield under drought (*qDTY*_*12.1*_)^14^ from crossing an *aus* and an *indica* rice genotype, i.e. Vandana and Way Rarem, respectively. Vandana is drought tolerant variety in Indian upland ecosystems and can produce some yield under severe drought. Way Rarem is a high yielding Indonesian rice variety that is susceptible to drought. Two well proven aspects of this QTL currently make it particularly unique. First, *qDTY*_*12.1*_ from Way Rarem increases the yield advantage of an already drought tolerant genotype, Vandana, under reproductive stage drought^14^. Second, *qDTY*_*12.1*_is valid in multiple genotypes^14^,^15^, multiple ecosystems^9^,^15^ (upland and lowland) and in multiple edaphic and climatic environments of different countries^9^,^15^,^16^,^17^. Meta-analysis of grain yield QTL identified under drought in grasses showed the presence of *qDTY*_*12.1*_ in 85% of the cases, the highest of all QTLs^9^. The present study was thus undertaken to understand the molecular factors underpinning such a versatile and stable QTL.

Previous results that formed the backdrop of the present undertakings were an increased water uptake capacity of the QTL^+^lines^18^ and the fractionation of *qDTY*_*12. 1*_ in two sub-QTLs^19^. Based on these results, the driving hypotheses were that *qDTY*_*12.1*_ may affect root growth and that there would be more than a single gene underpinning the functionality of *qDTY*_*12.1*_. To test these hypotheses, near isogenic lines (NILs) of *qDTY*_*12.1*_were generated and characterized. We identified and validated the transcription factor ‘no apical meristem’ (*OsNAM*_*12.1*_) as the mainstay of *qDTY*_*12.1*_. The *qDTY*_*12.1*_NILs exhibited increased root and panicle branching, transpiration efficiency, and yield under drought. Transgenic rice plants overexpressing *OsNAM*_*12.1*_ exhibited partial recapitulation of these responses and implied unaccounted factors. Explicably, promoters of six intra-QTL genes contained *NAM* binding-sites. Three more co-localized genes were putative functional partners or were at least co-expressed with *OsNAM*_*12.1*_. QPCR, EMSA and transcriptome analysis supported a central role for *OsNAM*_*12.1*_ in concert with the co-localized genes. Such a multigenic nature of *qDTY*_*12.1*_was confirmed through further analyses of transgenic plants overexpressing or complementing *OsNAM*_*12.1*_, intra-QTL recombinants, and insertion mutants. The effectiveness of *qDTY*_*12.1*_ in multi-environment field tests was thus rationalized by our results. This novel report on extensive molecular characterization of a QTL contributed by a susceptible variety that improves stress tolerance, as well as the identification of *cis*-interacting genes belonging to independent GO-terms within a QTL highlights the complex molecular interactions behind complex traits such as grain yield under drought. Further elaboration of the function and deeper crosstalk of these genes remain to be conducted but together they most likely form the basis for the stability and versatility of large-effect *qDTY*_*12.1*_. Similar identification and analyses of large-effect QTLs, combined with gene-based marker-assisted-precision breeding for complex traits, might fast-track crop improvement.

## Results

### Generation and characterization of qDTY_12.1_ NILs

**Figure 1:**
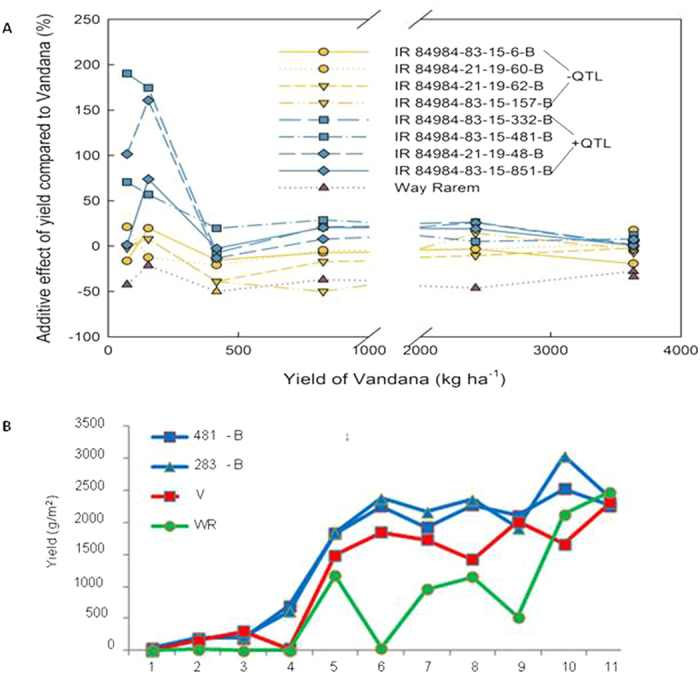
Effect of q*DTY*_*12.1*_ on yield under drought. (**A**) Additive effect of *qDTY*_*12.1*_ QTL+ and QTL- lines for grain yield over Vandana under varying severity of drought stress in six experiments conducted over three seasons at IRRI. (**B**) Grain yield data for the parental lines Vandana and Way Rarem and two NILs, 481-B and 283-B, from 11 trials over three years. NILs consistently outperformed the recipient parent Vandana for yield under drought.

**Figure 2:**
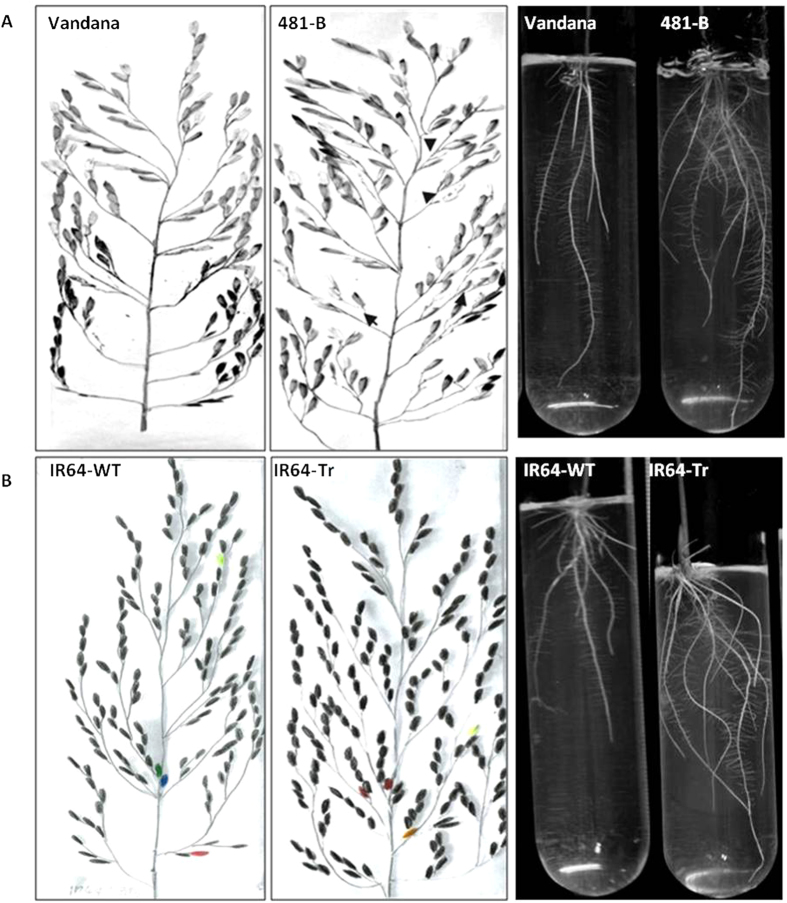
Panicle and root branching. (**A**) Under reproductive stage drought, 481-B exhibited increased numbers of secondary branches, total spikelets and filled spikelets in the panicles as a route to increased yield compared to Vandana. Similarly root branching was also increased in 481-B under PEG 8000 simulated water deficit as seen here as well as in the field (Figure S7). (**B**) IR64-Tr-E2 plants overexpressing the *OsNAM*_*12.1*_ recapitulated similar traits of panicles and roots under drought but with reduced effect on yield compared to 481-B.

NILs of Vandana with *qDTY*_*12.1*_were developed using a marker assisted backcrossing scheme (Figure S1). These NILs carried the Way Rarem *qDTY*_*12.1*_ allele with 93.4 to 95.9% recovery of the Vandana genome (Table S1 , Figure S2). Two sets of field studies were conducted for characterization of the *qDTY*_*12.1*_; BC_2_-derived lines were tested in six trials across three seasons, and another 11 trials were conducted across six seasons with the selected NILs. The average additive effect of *qDTY*_*12.1*_ on grain yield ranged from 4% in well-watered conditions to 104% under severe drought (means of additive effect from Fig. 1). No significant differences between Vandana and the NILs were observed in terms of yield and yield-related traits, under non-stress conditions (Table S1). However, under drought, the NILs had the following distinguishing features from the recipient parent Vandana: 300–600 kg ha^−1^ more grain yield, with the best performing NIL, IR84984-83-15-481-B (481-B), 25 times better than Vandana (693 compared to 27 kg ha^-1^); increased height, biomass, and harvest index (Fig. 1 and Table S1); and increased secondary branching of the panicle, concomitant with an increased number of filled grains per panicle (Fig. 2A and S4). The performance of Vandana (recipient), Way Rarem (donor), and the NIL 481-B were visually distinguishable under reproductive-stage drought in field conditions (Figure S3A); NIL 481-B flowered and set grains while Way Rarem did not, and Vandana exhibited a few panicles but much less than 481-B. After the NILs were fixed and showed no further ‘within line’ segregation, NIL 481-B showed similar grain type to that of the recipient parent Vandana (bulk plot harvest), (Figure S3D). The NILs also showed increased drought tolerance at the seedling stage, measured as an increase in shoot growth and root branching (Figure S5). Transpiration efficiency (TE) under drought was consistently higher in 481-B than Vandana through each of four different methods used for its measurement (Figure S6). NIL 481-B had increased root branching *in vitro* under PEG-simulated water-deficit (Fig. 2A), and this increased root branching was evidenced by lateral root growth under drought in the greenhouse (Figure S7A) and in the field (Figure S7B). These results supported the yield advantage of *qDTY*_*12.1*_ under drought and suggested 481-B as the NIL appropriate for further studies.

### Identification of candidate genes

**Figure 3:**
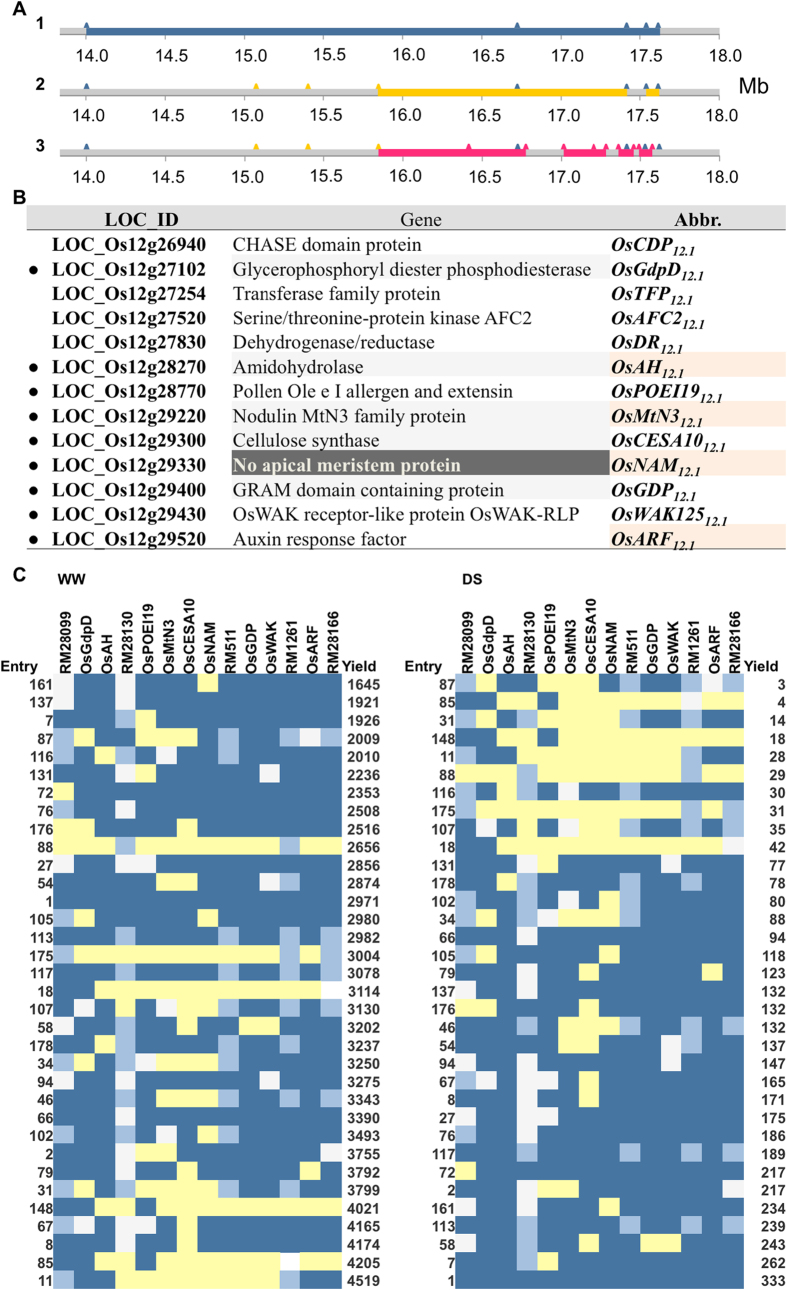
Identification of gene-based high density markers and candidate genes. (**A**) Schematic representation of the q*DTY*_*12.1*_ region for advancement in marker saturation. 1. Five markers (blue peaks) used for the original QTL detection. 2. A further 3 markers (orange peaks) for fine mapping detected two sub-QTLs. 3. Nine more gene-based markers (red) used in MCMC analysis detected 4 sub-QTLs. The original QTL was reduced from 3.6 Mb to 1.8  Mb to 1.5 Mb successively. (**B**) Genes used as gene-based markers (dotted) by a strategy outlined in Figures S8, S9 and Table S2. Grey shade indicates putative *OsNAM*_*12.1*_ targets. Final mapping shifted the QTL peaks directly to four putative candidate genes (beige) based on results in C. (**C**) Heat map style representation of the result that higher the number of Way Rarem alleles, more improved the yield. Raw data represented for yield (Y-axis, right; kg ha^−1^) in recombinant lines (Y-axis, left) tested for 14 markers (X-axis, top) under well watered (WW) and drought stress (DS) conditions. Alleles are indicated by colors: yellow = Vandana; blue = Way Rarem; light blue = heterozygous, and white = unknown.

Identification of candidate genes was based on the hypothesis that multiple genes underlie *qDTY*_*12.1*_ functionality because the original 3.6 Mb QTL region^14^ reduces to 1.8 Mb and fractionates into two sub-QTLs of 0.5 and 0.07 Mb with distributed yield advantage^19^. Of these, only the 0.5 Mb sub-QTL was detected in a metaQTL analysis^9^. For high-density mapping around and between the peak markers of the sub-QTLs and for simultaneous identification of the putative candidate genes, the 1.8 Mb DNA was sequenced. Excluding the (retro)transposons, expressed and hypothetical proteins, 53 gene models were compared between Vandana and Way Rarem. More SNPs and indels were noted within the larger sub-QTL (Figure S8) where a *cellulose synthase* gene (LOC_Os12g29300; Os*CESA10*_*12.1*_) was completely absent in Vandana. Genevestigator-mediated values for fold-change in gene expression under drought, when combined with the number of SNPs, revealed 30 *qDTY*_*12.1*_ genes with major differences between Vandana and Way Rarem (Figure S8, selection 1). *In silico* data and literature mining revealed that among the 30 genes, 17 were co-expressed with two or more other drought-responsive genes (Figure S8, selection 2). After further *in silico* analysis for promoter *cis*-regulatory elements, protein-domains, and protein partners responsive to drought, 13 putative candidate genes were shortlisted (Figure S8, selection 3; Fig. 3A,B). Ten of these genes (containing the central yellow dot in Figure S9) were chosen for further analysis due to either putatively direct regulation by *OsNAM*_*12.1*_ or a direct link to strongly inducible drought responsive transcription factors (Figure S9). The gene *OsWAK*_*12.1*_ was an exception and was chosen based on strong evidence from the literature for its function highly relevant to stress tolerance^20^. Nine of these 10 genes spread along *qDTY*_*12.1*_ were finally used as gene-based markers in combination with the five original SSR markers to ensure marker collinearity at an average marker distance of about 0.2 cM for high-density mapping (Fig. 3B). Such a fine mapping strategy ensured simultaneous progress on identification of the candidate gene underpinning *qDTY*_*12.1*_. Nearly 1900 BC_2_F_3_ lines (Figure S1, red box) were genotyped with the five SSR markers along *qDTY*_*12.1*_. This led to the identification of 33 intra-QTL recombinant lines, which were further analyzed with 14 collinear markers (5 SSR +9 gene-based). This analysis revealed that a higher number of Way Rarem alleles along *qDTY*_*12.1*_ corresponded with a higher yield under drought (Fig. 3C). This result supported a role for multiple *qDTY*_*12.1*_ genes in yield under drought. Bayesian MCMC (R/qtlbim) analysis of this data fractionated *qDTY*_*12.1*_ into 4 regions (Figure S8B) and suggested genes underlying the four LOD peaks as the four putative candidate genes; an amidohydrolase, a nodulin/SWEET13, no-apical-meristem, and an auxin response factor (*OsAH*_*12.1*_, *OsMtN3*_*12.1*_, *OsNAM*_*12.1*_ and *OsARF*_*12.1*_, respectively). The four genes together accounted for 21.9% of the heritability, although only *OsMtN3*_*12.1*_ and *OsNAM*_*12.1*_ lay around the original LOD peak subtended by the marker RM511 in the 36 Mb QTL. Thus the choice for the strongest candidate gene was between *OsMtN3*_*12.1*_ and *OsNAM*_*12.1*_.

### Molecular basis for multiple candidate genes

The four genes, *OsAH*_*12.1*_, *OsMtN3*_*12.1*_, *OsNAM*_*12.1*_ and *OsARF*_*12.1*_ were putative functional interactors with other nine genes in a manner suggestive of a drought-responsive local co-expression hub centred on *OsNAM*_*12.1*_ (Figure S9; Table S2). Promoter *cis*-regulatory element analysis had predicted the presence of *NAM/NAC* binding sites CATGTG, TTNCGTR, and TTNCGTRrc in six genes, including the four putative candidate genes (Table S3 and Figure S9). Several binding sites described by Jensen and Skriver^21^ were used to build the latter two degenerate consensus motifs. Recombinant OsNAM_12.1_ binding to promoter fragments of all six genes was confirmed through the electrophoretic mobility shift assay (EMSA; Figure S10). The EMSA results demonstrated variable extent of binding of the recombinant OsNAM_12.1_ to the specific competitor, despite similar experimental conditions. This was reflected in variable intensities of the band shift (lane 2, Figure S10), which arose due to variable amounts of free r-OsNAM_12.1_ available to bind to the specific hot probe. The specific binding increased as expected in the absence of the competitor (lane 3, Figure S10).

Quantitative real-time PCR (qPCR) analysis (Figure S11) revealed that, except for *OsWAK*_*12.1*_, the trends in up- or down-regulation of the nine genes under drought were the same in Vandana and Way Rarem. However, the strength of such regulation was different in the two genotypes for some genes such as *OsAH*_*12.1*_, *OsPOEI19*_*12.1*_, *OsMtN3*_*12.1*_ and *OsNAM*_*12.1*_ (Figure S11). The drought-responsive trend was reversed only for *OsNAM*_*12.1*_, from a slight down-regulation in Vandana and Way Rarem to an appreciable up-regulation in 481-B. In 481-B, the *OsNAM*_*12.1*_ content was relatively less than either parent under well watered conditions but more than either parent under drought. Thus, both the expression trends and the content of *OsNAM*_*12.1*_ are affected in 481-B, further justifying the selection of *OsNAM*_*12.1*_ as the critical candidate gene. Such alterations in the expression trends or content of these genes in 481-B compared to Way Rarem in well watered or drought conditions suggested *trans* effects of the Vandana genomic background. This is the case for *OsGdpD*_*12.1*_, *OsAFC2*_*12.1*_, *OsPOEI19*_*12.1*_, *OsGDP*_*12.1*_and *OsARF*_*12.1*_. If expression patterns of the nine genes in 481-B are considered ideal for the drought tolerant morpho-physiology, then *OsNAM*_*12.1*_, *OsGDP*_*12.1*_ and *OsPOEI19*_*12.1*_ are up-regulated, *OsAH*_*12.1*_, *OsAFC2*_*12.1*_, *OsMtN3*_*12.1*_ and *OsARF*_*12.1*_are down-regulated, and *OsGdpD*_*12.1*_ and *OsWAK*_*12.1*_ show no change. In terms of drought response, 481-B could be a conditional over-expresser of *OsNAM*_*12.1*_ whereby its putative target genes assayed by EMSA should also be conditional over-expresser in 481-B compared to Way Rarem. Four of the six targets assayed by EMSA do show such an upregulation under drought in 481-B. *OsMtN3*_*12.1*_ was an exception and *OsCesA10*_*12.1*_ was not analyzed due to the lack of this gene in Vandana. The high-density mapping, EMSA, and qPCR results together reinforced the hypothesis that multiple genes are involved in *qDTY*_*12.1*_ functionality. Additionally, these results led to the hypothesis that *OsNAM*_*12.1*_ may be at the centre of a concerted response to drought.

### OsNAM_12.1_ over-expression recapitulates 481-B drought response

To validate the apparently central role of the Way Rarem *OsNAM*_*12.1*_, it was over-expressed in the drought susceptible rice genotype IR64. Three independent single copy events were analyzed at the T3 homozygous state. Transgenic plants (IR64-Tr) exhibited increased root and panicle branching and transpiration rate (Fig. 2B and S12). The number of root branches in event 1 was similar to that of the WT, but events 2 and 3 showed significantly more root branching than the WT (Figure S12A). Similarly, the number of secondary branches per panicle varied in the different events (Figure S12B) but was significantly different from the IR64-WT. Such variability among transgenic events is common and mostly related to position effects, but also may occur because of differences in spatio-temporal metabolic fluxes whereby the effect of a single gene, especially those involved in quantitative traits, may be masked by differential interactions between the proteins and/or metabolites. Nonetheless, event 2 showed increased branching in both roots and panicles and higher transpiration rates under drought compared to the WT, a phenotype similar to 481-B. Therefore, event 2 was chosen for further studies. Comparison of IR64-Tr-E2 with IR64-WT for panicle characteristics clearly established significant increase in the number of total spikelets and fertile spikelets in the transgenic plant (Figure S12D). Under drought its yield increase over the WT was nearly half that of 481-B over Vandana (‘SF’ in Figures S4 and S12D). Similarly, increase in root and panicle branching in IR64-Tr-E2 over IR64-WT lagged behind the increase in 481-B over Vandana and suggested a positive but sub-optimal effect of *OsNAM*_*12.1*_, once again supporting the multi-gene hypothesis.

### Root transcriptome comparison

To understand how *OsNAM*_*12.1*_ over-expression influences the target genes within *qDTY*_*12.1*_and across the genome to affect root growth, the root transcriptome of Vandana, 481-B, IR64-Tr-E2, and IR64-WT were compared under control and drought conditions. This revealed differential expression of 7674 genes (Figure S13A-C). For IR64-WT *versus* IR64-Tr-E2 under stress, 553 genes were distinctly regulated (Figure S14; Table S4). Gene sets that were more up-regulated in the transgenic roots under drought were related to auxin, brassinosteroid and ABA signal transduction; WRKY-mediated transcription regulation; mitochondrial electron transport; redox regulation (glutathione S-transferases); transport regulation (aquaporins) and wall associated kinase complexes (Table S4). Conversely, stress responsive and acclimation related genes such as cell-death specific cysteine proteases, LEAs, dehydrins, heat-shock proteins, oxidoreductases and stress-associated transcription factors of the Myb, HSF and C3H zinc finger protein family were more up-regulated in the WT roots (Table S4). A curtailed reaction to stress by the transgenic roots may be a cause or effect of regular function, as implied by the upregulation of genes for better water uptake and transport and energy sufficiency. Such a response most likely led to their better homeostatic/physiological status for continued but modified growth under stress as opposed to the impeded growth of WT roots.

Within *qDTY*_*12.1*_, 38 genes were differentially regulated under drought. Six of the initially shortlisted 13 genes (*OsCDP*_*12.1*_*, OsDR*_*12.1*_*, OsWAK125*_*12.1*_*, MtN3*_*12.1*_*, GDP*_*12.1*_, and *OsARF*_*12.1*_) were prominently up-regulated (Figure S15). These included the EMSA-validated targets of *OsNAM*_*12.1*_ and/or qPCR-validated drought responsive genes. Other genes putatively *trans*-regulated by *OsNAM*_*12.1*_ included members of gene families, representatives of which were implicated as *cis*-regulated by *OsNAM*_*12.1*_ in *qDTY*_*12.1,*_ for example: GDP, AFC2, POLE, CESA, MtN3, NAM and WAK. Importantly, expression of *qDTY*_*12.1*_genes compared between IR64-Tr-E2 and IR64-WT under drought largely mirrored the differences in expression of these genes compared between Vandana and 481-B (Figure S15). For most genes, however, IR64-Tr-E2 exhibited higher scales of regulation (positive or negative) compared to 481-B under drought. This suggested the effect of *OsNAM*_*12.1*_ and supported it as a centrally important gene of *qDTY*_*12.1*_. Retrotransposons within *qDTY*_*12.1*_(CACTA, TY3-gypsy subclasses) were more prominently suppressed in the transgenic roots under stress. Their functional significance, and relationship to *OsNAM*_*12.1*_ under drought, if any, requires further research. Nevertheless, the transcriptome analysis reiterated possible roles for multiple genes of *qDTY*_*12.1*_ for its optimal functionality while highlighting putative *trans*-genomic and *cis*-*qDTY*_*12.1*_ gene sets affected by *OsNAM*_*12.1*_.

### Complementation of OsNAM_12.1_ in Vandana

**Figure 4:**
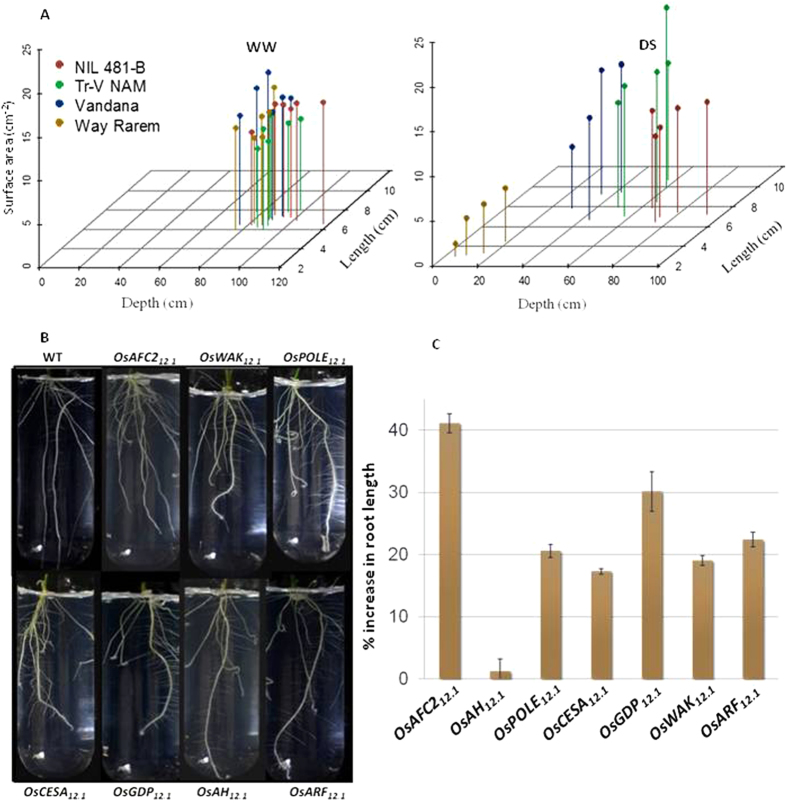
Root analysis of the transgenic plants. (**A**) 3D scatterplot for total root traits in Vandana, Way Rarem, 481-B, and transgenic Vandana overexpressing *OsNAM*_*12.1*_. Data was collected with ImageJ root analyzer ((http://imagej.nih.gov) on 2 week old plants with 8 to 15 plants per genotype under well watered (WW) and drought stress (DS) conditions. Statistical analysis and graph plotting were performed using R software (http://CRAN.R- project.org/package=scatterplot3d). Multiple stalks of a single color represent mean values for multiple samples within a ‘genotype’, which represent different standard deviations within the group for the three root traits. (**B**) Panel showing that plants mutated for the particular genes led to higher lateral root growth than the WT. (**C**) Quantitative difference in total root length of each mutant as percent increase over WT. Up to five seedlings from pots were sampled for each mutant and WT.

The Vandana allele of *OsNAM*_*12.1*_ was different from the Way Rarem allele in three amino acids, K7N, S109N, and insertion of a P at 223, while the promoter of the Vandana allele lacked a 246 bp indel with critical drought responsive *cis*-regulatory elements (Figures S16 and S17). Due to these alterations, the Vandana allele might be a functional knock-out despite its RNA and protein expression. Thus, its possible complementation was assessed by over-expressing the Way Rarem allele in Vandana. These transgenic plants, especially the line V580, exhibited marginally increased root growth under normal conditions but extensively increased root growth under drought (Fig. 4). The three transgenic lines of Vandana were relatively shorter and sterility was more than in the WT plants. For this reason panicle branching or yield data were not comparable. Constitutive over-expression of NAM/NAC transcription factors is often known to lead to dwarf plants^22^, which was also observed with some transgenic lines of IR64. Sub-optimal effects of *OsNAM*_*12.1*_ on its putative target traits in IR64 and Vandana, compared to when it is introgressed as part of *qDTY*_*12.1*_, reiterated the need for Way Rarem alleles of additional *qDTY*_*12.1*_ genes. NAM/NAC genes regulate various genes, which in turn affect biochemical pathways and physiological mechanisms in different crops in response to biotic, abiotic and developmental cues^21^. The effect of NAC genes on drought tolerance in rice by root architecture modification is known^23^,^24^. However, the phylogeny and protein domain structure of *OsNAM*_*12.1*_ makes it rather unique (Figure S16), perhaps leading it to regulate unique sets of genes which might underlie the *OsNAM*_*12.1*_ effects on multiple traits under drought.

### Role of additional qDTY_12.1_ genes

In order to assess the importance of the additional genes implicated for a role in the functionality of *qDTY*_*12.1*_, insertion mutants were identified in the TRIM database^25^ for 7 of the 13 genes. Insertion-site flanking-sequence-analysis-mediated single site insertion-mutants were grown to obtain plants homozygous for the insertion. *OsAH*_*12.1*_ was evaluated as a knock-out (KO) mutant while the six other genes (*OsGDP*_*12.1*_, *OsAFC2*_*12.1*_, *OsPOLEI19*_*12.1*_*, OsCESA10*_*12.1*_, *OsWAK*_*12.1*_ and *OsARF*_*12.1*_) were evaluated as activation tag mutants (AT). All seven mutants exhibited increased root branching compared to the wild type in tube and pot studies (Fig. 4B, C). Increased root branching in the *OsAH*_*12.1*_-KO line supported the concept that it was a negative regulator of root branching; while root branching increased in 481-B under drought, it exhibited down-regulation of *OsAH*_*12.1*_ (Figure S11). As a corollary, other down-regulated genes such as *OsAFC2*_*12.1*_and *OsARF*_*12.1*_may also be negative regulators and hence their AT lines should exhibit reduced root branching. However, increased root branching of the *OsAFC2*_*12.1*_and *OsARF*_*12.1*_AT lines suggested that the role of these genes in 481-B may be under more complex genetic/genomic controls, not least of which might be the influence of altered expression trends and content of *OsNAM*_*12.1*_. Similarly, *OsWAK*_*12.1*_expression in 481-B did not change much under drought, yet the AT line showed increased root branching. On the contrary, increased root branching in the AT lines of other genes suggested more straightforward regulation since their expression under drought in 481-B was also up-regulated. These results in combination with the EMSA assays suggested that *OsNAM*_*12.1*_could be a positive or a negative regulator, similar to another drought-responsive transcription factor DREB2A^26^. The links depicted in the schematic Figure S9 suggest possibilities of crosstalk between the genes. For example, feedback and feed-forward loops may be possible in the case of *OsAFC2*_*12.1*_and *OsPOEI19*_*12.1*_ linked to *MYB* transcription factors and *OsNAM*_*12.1*_linked to the WRKY transcription factor. WRKY genes are extensively auto/cross regulated under stress^27^ and the *MYB* genes are feedback-regulated by their downstream target miRNA genes^28^. Similarly, direct or indirect putative links between the genes, illustrated by the blue lines connecting the green circles in Figure S9, may also influence each other’s expression. Such crosstalk among *NAM/NAC*, *MYB*, *WRKY*, and *CesA* genes has been well documented for the process of secondary cell wall formation^29^. Such intra-QTL crosstalk and genomic background difference/interaction might underlie variations in gene expression and trait phenotypes when 481-B, V580, and IR64-Tr-E2 are compared.

## Discussion

Altered root architecture and transpiration efficiency are related to drought tolerance^30^ occasionally through NAM/NAC family transcription factors^31^. However, our results implicated the concerted action of *OsNAM*_*12.1*_ in *qDTY*_*12.1*_ in novel traits. For example, increased secondary branching of the panicles and concomitantly in the number of spikelets under drought might be an important component of yield under drought. In addition to its known validity in multiple genotypes, environments, and ecosystems^32^, *qDTY*_*12.1*_ was valid not just at the reproductive stage but also at the seedling stage (Figure S5) making it a highly valuable QTL.

The variation noted among the NILs in Figure S5 was not due to background effects; all NILs were developed from one F3-derived line and two BC2-derived sister lines whose backgrounds were different only in one marker among all of the markers employed. A reason for variation among NILs could be the micro-heterogeneity common to drought experiments and quantitative traits. Another reason could be the gene x gene (G × G) interactions as seen through the metabolite and proteome analysis of *qDTY*_*12.1*_ NILs in comparison with the parental genotypes^33^,^34^. Such ‘interactions’ may assume higher proportions of variability that lead to differences among the NILs given the fact that *qDTY*_*12.1*_ was contributed by the susceptible parent Way Rarem, making the tolerant recipient parent Vandana even more tolerant. These implicated epistatic interactions may not be spatio-temporally constant due to metabolic fluxes. Such epistasis was documented earlier, and *qDTY*_*12.1*_ was shown to be epistatic to two QTLs from Vandana, i.e. *qDTY*_*2.3*_ and *qDTY*_*3.2*_^35^. This epistatic effect on gene expression was particularly observed on *OsNAM*_*12.1*_, where a drought-mediated down-regulation in parental and WT plants changed to an up-regulation under drought in the NIL 481-B and in transgenic plants of Vandana and IR64 overexpressing *OsNAM*_*12.1*_ (Figure S11). Additionally, the yield under drought was greatest when the entire QTL region was of the Way Rarem type (Fig. 3C). However, other intra-QTL gene combinations were also valid for increased yield under drought, implicating intra-QTL epistasis, which we detected on analysis of the data in Fig. 3C. Hence, the two levels of epistasis can be hypothesized to underlie variation in the performance of the NILs. Moreover, the fact remains that Vandana never performed as well as most of the NILs for yield under drought and allied traits, including shoot and root biomass. Thus it can be accepted that the features observed in the NILs were due to *qDTY*_*12.1*_ and not due to their minor differences in the genomic background.

Results on morpho-physiological characterization of the NIL 481-B taken together with the previous observation that *qDTY*_*12.1*_ increases water uptake under drought^18^ suggested that *qDTY*_*12.1*_ responded via developmental and physiological adaptations. Results of field tests under different soil types and different severity of drought^15^,^17^,^32^ suggest a complex, multi-trait-based mechanism driving *qDTY*_*12.1*_. This is borne out by the comparative primary metabolites and proteome analysis of 481-B, which reveal extensive changes in the content of sugars and amino acids in the roots and flag leaves under drought but not in the spikelets^33^,^34^.

A single SNP is enough to lead to a new phenotype, mostly if that is a non-synonymous change altering an amino acid. A single SNP altering a trans-acting protein binding site in the promoter can also be causal to a phenotype. Through our sequencing results we noted numerous changes in most of the genes when the *qDTY*_*12.1*_ region was compared between the parental lines Vandana and Way Rarem, highlighting the possible role of SNPs in non-synonymous changes in amino acids or in particular protein binding elements of the upstream sequences. Since genes that do not show SNPs between Vandana and Way Rarem had almost no chance of being a candidate gene, the first level of selection was only to state that all the genes that have SNPs are likely to be candidate genes. Those that had SNPs but whose expression was not influenced by drought, as judged through Genevestigator, did not go to the next stage of selection.

Gene expression of the nine interlinked genes was compared in the roots of the two parental (V and WR), NIL (481-B), WT (IR64) and transgenic plants (V580 and IR64-Tr-E2) (Figure S11). *OsNAM*_*12.1*_ was indeed over-expressed, as expected in the transgenic V580 and IR64-Tr-E2 but also in 481-B. Given the regulation of the five genes (whose promoter binding is documented in Figure S10) by *OsNAM*_*12.1*_ (*OsCesA10*_*12.1*_ was not compared as Vandana lacks the gene), as well as the mutant analysis for root proliferation (Fig. 4), the *OsAFC2*_*12.1*_ and *OsARF*_*12.1*_ should be up-regulated and *OsAH*_*12.1*_ should be down-regulated under drought in the three tolerant genotypes, i.e. 481-B, V580, and IR64-Tr-E2. Under drought *OsAH*_*12.1*_ is down-regulated in 481-B and V580 and the upward trend of IR64-WT is curtailed in IR64-Tr-E2, effectively amounting to comparative down-regulation. *OsAFC2*_*12.1*_ and *OsARF*_*12.1*_ are unexpectedly down-regulated in 481-B but their expression increases or remains similar under drought in V580 and IR64-Tr-E2. Overall, V580 and IR64-Tr-E2 exhibited similar expression trends for five of the nine candidate genes under drought, and these were largely similar to expression trends in 481-B. However, *OsAFC2*_*12.1*_, *OsMtN3*_*12.1*_*, OsWAK*_*12.1*_and *OsARF*_*12.1*_ exhibited differential expression, which was reminiscent of the genomic and intra-QTL epistatic interactions. Most genes in transgenics V580 and IR64-Tr-E2 (with the odd outlier) follow the expression trends of 481-B in response to drought, suggesting their optimal role in the drought responsive morpho-physiological changes conferred by *qDTY*_*12.1*_. Largely similar yet slightly different gene-combinations of the nine genes could result in similar responses (Fig. 3C). These aspects highlight the complex gene interactions underlying the multigene quantitative trait of drought tolerance, and our results demonstrate this complexity within a field-validated QTL with a major effect on yield. Thus, a gene-cluster subtending linked sub-QTLs has now been illustrated.

Linked, interacting, or fractionating QTLs are known^36^,^37^,^38^,^39^. Such fractionating QTLs that are functional through clustered and/or multiple genes of different gene ontologies (multi-GO) are advocated^40^ and hypothesized^41^,^42^ and the underlying clusters have been predicted *in silico*^43^,^44^,^45^. However, multi-GO genes predicted for the functionality of any plant QTL have never been validated. Similarly, genes in the functional gene clusters representing gene families^46^, biosynthetic pathways^47^ or certain traits^48^,^49^ belong to a single GO category. The plant self-incompatibility (SI) S-locus is a unique case of multi-GO locus in which SI is governed in different plant families through genes of different GO categories, i.e. the S-locus receptor kinase and S-locus cysteine rich protein (SRK/SCR) in *Brassicaceae*; S-RNase and S-locus F-box proteins (SRN/SLF) in *Solanaceae/Rosaceae;* and the pistil S-determinant (calcium signaling) and a transmembrane protein, (the pollen S-determinant; PrsS/PrpS) in *Papaveraceae*^50^,^51^. However, these cases of two alternative genes are different from the multigene, genetic architecture of a regulon implied by the drought-mediated response of several multi-GO genes regulated by *OsNAM*_*12.1*_. The translation initiation factor eIF4E is proposed as a central node of an RNA regulon coordinately orchestrating the expression of multiple genes at the post-transcriptional level^52^, but no DNA regulons for clustered genes are known in eukaryotes. Our results do not establish *qDTY*_*12.1*_ as a regulon definitively, but do suggest the potential for such a genetic architecture, which must be proven in the future through further analysis.

Due to their different GO categories, the potential roles of the genes in *qDTY*_*12.1*_putatively regulated by *OsNAM*_*12.1*_ span a variety of functions (Table S2). Two genes, *OsAH*_*12.1*_ and *OsAFC2*_*12.1*_, potentially have the ability for global transcriptome modulation through affecting DNA methylation and alternative splicing, respectively. *OsAH*_*12.1*_ is highly similar to a deaminase for s-adenosyl homocysteine (SAH), which is a potent inhibitor of DNA methyltransferases^53^, and *OsAFC2*_*12.1*_ is highly similar to a kinase for phosphorylation-mediated S/R protein activation as a component of the spliceosome (Table S2). However, the putative co-expression and crosstalk connections illustrated in Figure S9 implicate a role for all nine intra-QTL genes, which are further connected to important drought/stress responsive genes including WRKY transcription factors. *OsMtN3*_*12.1*_ is *Xa25*^54^, which was also identified as the *SWEET13*, and its homologs *SWEET11* and *SWEET14* were shown to be hexose sugar transporters^55^ which can have a critical role in drought tolerance^56^. Of the 13 rice *OsGdpD* genes, preliminary results implicate *OsGdpD*_*12.1*_ specifically in the elongation of anther filaments and root hairs, both of which may be important factors to counter reproductive stage drought. In white lupin, two *GdpDs* were shown to be associated with root hair development and phosphorus deficiency tolerance^57^, which may be linked to drought response due to reduced nutrient diffusion under drought^58^,^59^. Similarly, an Arabidopsis Gram Domain Protein (GDP; a likely ortholog of *OsGDP*_*12.1*_) was shown to be ABA- and stress-responsive and affected reproductive development^60^. A rice homolog of *OsWAK*_*12.1*_ was also associated with female gametophyte development^61^, while silencing of another *OsWAK* led to abnormal root development and anther indehiscence^20^. *OsPOEI19*_*12.1*_ has high similarity to an Arabidopsis ortholog (AT5GO5500) that is known to be functional in root hair elongation, while the cell wall forming cellulose synthases similar to *OsCesA10*_*12.1*_ are also known to be functional in root and root hair development^62^. Apart from these genes, the root transcriptome comparison between Vandana and 481-B under drought revealed upregulation of an AAA-type ATPase (LOC_Os12g28550) within *qDTY*_*12.1*_. ATPases couple energy generation with macromolecule translocation^63^ and are known to enhance drought and salinity tolerance^64^. With two pertinent transcription factors, two additional putative transcriptome modulators responsive to drought, and a suite of genes associated with root or reproductive organ development, often under drought, *qDTY*_*12.1*_ is well structured as a large-effect QTL for yield under drought.

For insertion mutation in the co-localized genes and their effects on root phenotype, the positive effect of *OsARF*_*12.1*_-AT on lateral root development was reiterative of the role of ARFs^65^ but opposite to that expected from qPCR and transcriptome analysis which showed *OsARF*_*12.1*_ down regulation in 481-B and transgenic V580 while remaining almost unchanged in IR64-Tr-E2 under drought (Figure S11, S15). These results can be expected because *OsARF*_*12.1*_, categorized as *OsARF24*^66^,^67^, may be a negative regulator like its Arabidopsis ortholog *AtARF2*^68^, and perhaps functionally similar to *OsARF12*, which is known as a negative regulator of inorganic phosphorus absorption and translocation and lateral root growth^69^. Extensive characterization of the TRIM mutants is essential to establish their individual roles and the value of their combinatorial contribution to root architecture and to the function of *qDTY*_*12.1*_. However, our results reiterate the complexity of drought tolerance and the advantages of the large-effect QTLs. Additional large-effect QTLs valid in multiple systems have since been identified^32^, and preliminary analyses of two such QTLs support a similar multi-GO cluster-based functionality (AKo, unpublished results). In one of these instances, the most favored candidate gene is another NAM/NAC TF, which seems to be a master regulator similar to *OsNAM*_*12.1*_. Extensive network analysis in robust systems such as rice and Arabidopsis can identify such master regulators as exemplified recently for the transcription factor *HYR*^70^.

A limitation in our report is the lack of assessment of multiple genes in independent transgenic plants, and perhaps in various backgrounds, which could further validate the interactions of the multiple intra-QTL genes. Additionally, most experiments such as the qRTPCR and transcriptome analysis were performed on the root tissue whereas it would be useful to compare similar data for leaves, stems, and panicles. We have already performed such comparative studies for metabolites and proteomes^33^,^34^ and are currently undertaking the transcriptome comparison. Futhermore, characterization of additional intra-QTL genes could improve our understanding of *qDTY*_*12.1*_ function. For example, we excluded *OsCDP*_*12.1*_ and *OsDR*_*12.1*_ from further analysis when moving ahead with a selection of 10 genes from among the 13 shortlisted, but both of these genes showed strong correlation to drought response based on the transcriptome data (Figure S9). Finally, the transcriptome data revealed an intra-QTL AAA-type ATPase that was differentially regulated in Vandana and 481-B under drought. This was not detected as a potential candidate gene by our strategy, most likely due to lack of SNP identification between its Vandana and Way Rarem alleles, which in turn may be a limitation of the depth (15x) of the sequencing data. However, the transcriptome data also revealed a similar expression differential for this ATPase under drought between IR64-WT and IR64-Tr-E2 overexpressing *OsNAM*_*12.1*_, suggesting that the ATPase may also be regulated by *OsNAM*_*12.1*_. Nevertheless, these transcriptome analysis results identified additional *cis*-genes, further supporting the multi-gene hypothesis. Such identification of markers at different functional levels based on omics and mechanistic understanding can facilitate precision breeding.

In summary, the involvement of more than a single cardinal gene in *qDTY*_*12.1*_ functionality was demonstrated by six lines of evidence: intra-QTL recombinants, qRT-PCR, EMSA, transgenic plants, root transcriptome, and insertion mutants. The results suggest a role for genomic and intra-QTL epistasis that should be explored further to ascertain the reasons for some unexpected results, for example the differences in gene expression of some of the *OsNAM*_*12.1*_ target genes in 481-B and V580. Exploring the contribution of epistasis will also help in further understanding the molecular mechanisms operative in 481-B for its benefits under drought. Our results reiterate a role for the NAM/NAC transcription factors in lateral root proliferation under drought and also implicate a novel role for *OsNAM*_*12.1*_ in increasing spikelet fertility and the number of secondary branches and spikelets in panicles under drought. This study elucidates the molecular nature of factors involved in a QTL donated by a susceptible variety, and demonstrates a gene-cluster of genes of independent GO-terms interacting in *cis* to affect a trait – both of which are novel contributions to our understanding of complex traits such as drought response. These results set the scene for identification of similar *cis*-acting multi-gene clusters with large effect on a trait. Already, additional drought-yield QTLs have been identified and their pyramiding is now supporting fast-tracked improvement of rice^32^. Finally, our results underscore the much espoused complementation of field-based breeding and whole-plant physiology with data mining, molecular biology and systems biology approaches to mitigate food scarcity, hunger and poverty through rice science^71^.

### Experimental procedures

#### Plant material

*qDTY*_*12.1*_ was identified in an F_3:4_ population derived from the cross Vandana/Way Rarem^14^. IR79971-B-102-B, one of the F_3_-derived lines was used as the donor for *qDTY*_*12.1*_. This line was backcrossed to Vandana to develop BC_2_- and BC_3_-derived populations and NILs as outlined in Figure S1. A set of contrasting +QTL and –QTL BC_2_F_3_-derived lines was used for yield and morpho-physiology studies, while the line 481-B was used for most molecular studies. The BC_2_F_3_ lines were also used to identify intra-QTL recombinants, which were then used for genotyping with 19 markers for high density mapping.

#### Reproductive-stage drought screening experiments

Field experiments were conducted from 2010–2013 at the International Rice Research Institute (IRRI), Los Baños, Laguna, Philippines, located at 14^o^ 13′N latitude, 121^o^ 15′E longitude, at an elevation of 21 m. Screening of BC_2_F_3:4_ and BC_3_F_3:4_ lines with *qDTY*_*12.1*_ developed through MAB, and subsequent AYTs and physiology studies, were conducted using an α-lattice or randomized complete block design along with Vandana, Way Rarem, in 2–4 replications of 1–4 row plots 1.5–3 m in length, 0.25 m row-to-row spacing and 2.0–2.5 g seed per linear meter. Fertilizer and crop management practices were followed as earlier^72^. Stress was initiated by withholding irrigation at 35 days after sowing (DAS), and irrigation supplied when the soil water tension fell below –50 kPa at 30-cm soil depth. Based on grain yield, the additive effect of a line


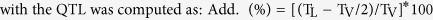


where Add. (%) is the percentage additive effect of the line with the QTL over the recipient parent (Vandana), T_L_ is the trait value for the line with the QTL, and T_V_ is the trait value of the recipient parent (Vandana).

#### Molecular breeding scheme

The MAB scheme for the transfer of *qDTY*_*12.1*_ into Vandana is shown in Fig. S1. Seeds from 62 different lines coming from BC_2_- and BC_3_-derived populations were multiplied under non-stress conditions and confirmed for the presence of *qDTY*_*12.1*_ along with background screening with segregating markers. Thirty-five selected lines with the *qDTY*_*12.1*_ segment were evaluated in advanced yield trials (AYTs) under upland stress and non-stress conditions and four BC_2_-derived and three BC_3_-derived lines with *qDTY*_*12.1*_, superior plant type, the highest Vandana genome recovery, and highest yield under non-stress conditions were identified. Additional information on drought screening and molecular breeding is provided in the Supplementary information.

#### Seedling stage trials

Seedling stage stress trials were established in upland fields in 2012 WS as described above. Drought was initiated at 7 DAS and plants were sampled to measure shoot biomass at 32 DAS. A seedling greenhouse study was conducted as described earlier^72^. Soil moisture treatments included well-watered (WW; maintained at field capacity) and drydown from field capacity (DD), with five replicates per genotype planted in an RCBD. Water uptake, shoot mass, and root length were determined as previously described^72^, including one nodal root from each plant with all lateral roots carefully spread apart in order to detect the number of root branches.

#### Generation of genotypic data

Rice SSR markers described earlier^14^ and three more (RM28076, RM28089, RM28099) were used for foreground, recombinant, and background selection on DNA extracted as described earlier^73^ from young leaves of 2-week-old plants. PCR was performed in 96-well plates as described^74^ and SYBR® Safe used to visualize DNA. The cM position described earlier^14^ was used for constructing chromosome maps, except for the three additional markers where the cM position was calculated based on the physical distance (Mb) of these markers from RM28048. Graphical genotyping software GGT2^75^ was used for the construction of chromosome maps of the selected lines. Nine additional gene-based markers were used (Fig. 3) for genotyping the intra-QTL recombinant lines.

#### Morpho-physiology measurements

Genetic variation for water uptake was determined by volumetric soil moisture at 10 cm depth increments (Diviner 2000, Sentek Sensor Technologies, Stepney SA, Australia). Root samples were taken at 58 (DAS) with 3 sub-replicates per plot using a 4-cm-diameter core sampler to a depth of 60 cm, washed, scanned, and analyzed as earlier described^76^. TE was assessed by carbon isotope analysis of the 2 youngest leaves sampled from 3 plants per plot at 2-week intervals from 21–70 DAS. Δ^13^C was calculated as

(-8-leaf ^13^C conc)

(1+(leaf ^13^C conc/1000))^77^

Instantaneous transpiration efficiency (TE) was determined at 44 DAS by LI-6400 portable gas exchange system (Li-Cor Inc., Lincoln, NE, USA). In all field trials, days to 50% flowering (DTF), mean plant height at maturity (PH), grain yield, and biomass were recorded as described earlier^78^ from a 2 m^2^ (stress) and 0.125–2.0 m^2^ (non-stress) area of each plot. Statistical analysis approach is outlined in Table S5. Statistical analyses for the physiology experiments were performed in R v. 2.8.0 (R Development Core Team, 2008) using ANOVA and LSD as the post-hoc test.

#### Root and panicle phenotyping

Mature dehusked seeds of all genotypes used were sterilized in 1% Sodium Hypochlorite and were germinated in autoclaved MS_0_ media pH to 5.8, in dark at 25–29 °C for 3 days. Ten pre-germinated seeds per line were transferred into MS_0_ with and without 10% (w/v) PEG-8000) in test tubes and were grown under light at 29 °C. The root morphology was observed after 8 days and was documented using Nikon D90 camera under diffused light. WinRhizo software (Régent Instruments, Quebec, Canada) was used for analysis of soil-grown root samples. Two-three week old seedlings were used for well watered or drought treated samples in pot based root phenotyping, and roots were assessed after reproductive stage drought in field based studies. Panicles were assessed for primary and secondary branches, total spikelet numbers, and filled spikelets after harvest from pots, screen-house field or open field. At least 3 panicles were samples from a minimum of 10 plants to facilitate usage of the data as a measure of yield.

#### SNPs in the 53 genes of qDTY_12.1_ region

Targeted sequencing of the QTL region from Vandana and Way Rarem genomes was performed with sequencing libraries using Agilent SureSelect protocol for paired-end Illumina platform (Agilent Technologies: SureSelectXT Target Enrichment System for Illumina Paired-End Sequencing Library, V1.4.1). Data processing, base calling, and extraction of cluster intensities were was done using RTA 1.12.4 (HiSeq Control Software 1.4.5). Sequence quality filtering script was executed in the Illumina CASAVA software (ver 1.8.2, Illumina, Hayward, CA). The raw Fastq reads were mapped/aligned to the IRGSP Build 5 (http://rgp.dna.affrc.go.jp/E/IRGSP/Build5/build5.html) reference sequence using the Bowtie2 (http://bowtie-bio.sourceforge.net/bowtie2/index.shtml) aligner. A few rounds of quality control were typically performed for removal of reads with poor quality: reads with average scores less than 30 and with ambiguous (N) bases, PCR duplicate artifact and reads outside the SureSelect region. Finally, SNPs were identified in Genespring 12.5.

#### Candidate gene selection

There were 118 genes in 1.75 Mb spanning the QTL after dropping the (retro)transposons. These were analyzed *in silico* for expression under drought from three experiments, (GSE26280, GSE24048 and GSE6901) at *ricearray.org*. Manual differential expression analysis was conducted in Microsoft Excel using the relative values; SAM module in Excel (stat.stanford.edu/~tibs/SAM), SAM module in TIGR MeV4 (*tm4.org/mev*), and Genevestigator (*genevestigator.com*) were used. There were 28 recurrent genes common to the 30 genes selected as a first pass based on the number of SNPs in these analyses. After a literature survey and *in silico* analyses for gene ontology (GO; medcomp.medicina.unipd.it/Argot2/index.php), co-expression partners (RiceFREND: http://ricefrend.dna.affrc.go.jp/), promoter *cis*-regulatory elements (PLACE: http://www.dna.affrc.go.jp/PLACE/ and PlantCARE: http://bioinformatics.psb.ugent.be/webtools/plantcare/html/), protein domains (pFAM: http://pfam.xfam.org/ and PROSITE: http://prosite.expasy.org/), and protein partners (STRING: http://string-db.org/), 13 putative candidate genes were chosen. In another analysis, putative NAM binding sites were predicted in the 53 genes of the QTL. The NAM binding motif was generated from the literature, and binding sites were predicted using an in-house perl script.

#### High density mapping

Nearly 1900 BC_2_F_3_ lines were phenotyped for drought tolerance through yield analysis and these lines were then genotyped using 5 SSR markers described earlier^14^. Thirty four of these lines were genotyped with 14 collinear markers (five SSR and 9 candidate gene-based markers) using primers designed (Table S6) after comparing the Vandana and Way Rarem sequences obtained from the NGS data.

#### Preparation of c-DNA for expression study

Standard Trizol mediated (Ambion, Austin, TX, USA) RNA isolation was conducted. The cDNA was synthesized using the ImProm-II Reverse transcription system (Promega Corporation, Madison, USA) as per the manufacturer’s instruction.

#### Real-Time PCR protocol (qRT-PCR)

A 10 μl reaction volume consisted of 1.0 μl of normalized cDNA, 5 μl of 2X SYBR green PCR master mix (Roche Diagnostics GmbH, Germany) and 0.4 μl of 10 mM primer for each primer pair. Reactions were run in triplicate in a 7500 Fast Real-Time PCR System (Applied Biosystems, Foster City, USA). Amplification conditions were 50 °C for 2′, 95 °C for 2′, 40 cycles of denaturing at 95 °C for 10” and a combined annealing and extension step at 60 °C for 30″, followed by a disassociation stage from 60 °C to 95 °C (melting curve analysis). The comparative threshold cycle (ΔΔCt) method was used to quantify the relative expression levels. Primers used are listed in Table S7.

#### Recombinant OsNAM_12.1_

Recombinant OsNAM_12.1_ was expressed in BL21 *E. coli* cells from a *Bam*HI and *Xho*I construct in pET28A(+) (Novagen) amplified from pCAMBIA_NAM using the primers NAMpETFor and NAMpETRev (Table S7). Transformed cells were induced with 1 mM IPTG at 25 °C and induced cultures were lysed in 20 mM sodium phosphate, 0.5 M NaCl, pH 7.4 by sonication. The soluble fraction was extracted by centrifugation at 1000 × g for 30’. Protein was obtained after Ni sepharose column chromatography (GE healthcare), by eluting with 20 mM sodium phosphate, 0.5 M NaCl, 500 mM imidazole, pH 7.4.

#### Electrophoretic Mobility Shift Assay

EMSA was performed using the LightShift Chemiluminescent EMSA Kit (Thermo Scientific, USA) on about 200–400bp promoter regions of target genes amplified with the primers in Table S7. DNA was end labeled with biotin (Thermo Scientific, USA), purification using QIAquick Kit (QIAGEN) and 20 fmol/reaction of the labelled DNA was incubated in binding buffer (10 mM Tris-HCl pH 7.5, 40 mM NaCl, 5% glycerol, 1 mM EDTA), BSA (6 μg), poly (dIdC) (1 μg) with 140 ng of OsNAM_12.1_HIS for 20′ at 30 °C and run on 4% native PAGE. Signal was detected according to the manufacturer’s protocol. Unlabeled fragment was used as specific competitor.

#### Microarray material, hybridization and data analysis

Plants for the microarray analysis were grown during the 2013 wet season in soil-filled cylinders in a screenhouse as described previously^72^. Seeds of Vandana, 481-B, IR64 and the *OsNAM*_*12.1*_ overexpression line were sown in 5 biological replicates in a randomized complete block design for both the well watered and drought stress treatments. Soil in the well-watered treatment was maintained at field capacity for Vandana and 481-B and at 1.2x field capacity for IR64 and IR64-Tr-E2, based on the adaptation of those genotypes. The drought stress treatment for all genotypes was a drydown from 75% of field capacity. Tissue samples from roots of five different plants of each genotype for each treatment were collected at 25 DAS. After collection, all samples were wrapped in aluminum foil and placed directly into liquid nitrogen before storing at −80 °C. The plant material was lyophilized and then ground to a fine powder with liquid nitrogen prior to analysis.

A pilot study was conducted to identify and omit oligos with potential for cross hybridization across gene family members from rice root tissues. Upon selecting oligos with best Agilent base composition score, a custom eArray was designed for rice cDNA sequences from 59,336 target probes and 60K Agilent rice microarray (in a 60 K × 8 plex format) by Oaklabs GmBH. All of the unigene sets have been annotated and functionally classified into MapMan functional categories. Probe preparation, labelling, hybridization, and scanning of microarrays were carried out as described earlier^79^. Total RNA was isolated from root tissues using the TRIzol reagent (Invitrogen) and RNAeasy columns (Qiagen). RNA concentration was measured using a NanoDrop UV-VIS spectrophotometer (Peqlab) and quality of RNA samples was evaluated using RNA Nano 6000 kit (Agilent) and Bioanalyzer (Agilent). The Low Input Quick Amp Labeling kit for One-color Microarray-Based Gene Expression Analysis was used for labeling of RNA samples (50 ng) using cyanine 3 (Cy 3) fluorescent dye following the manufacturer’s instructions (Agilent). The labeled cRNA samples were subsequently purified using RNeasy mini spin columns (Qiagen) according to the manufacturer’s protocol and the quantity was recorded using a NanoDrop spectrophotometer. Based on that data, the specific activity and yield of cRNA were calculated. 600ng of Cy 3-labeled, amplified cRNA with a specific activity of above 6 were used for subsequent hybridization following the steps of the One-color Microarray-Based Gene Expression Analysis protocol (Agilent). Hybridizations were carried out at 65 °C for 17 h. Slides were washed and scanned at high resolution of 2 microns using an Agilent DNA Microarray Scanner G2565CA (Agilent). The resultant microarray TIF images were processed to run batch extractions by choosing the appropriate grid using Agilent’s Feature Extraction Software version 11.0. The quantified feature text file was first analysed for quality using the Agilent QC chart tool. The qualified experiments were further processed; raw data (gene expression) was first quantile normalized and fold changes were calculated between transgenic and wild type samples for control and drought stress treatments. The differentially regulated gene sets (>2.0 folds, with multiple correction using Bonferronic method (P < 0.05) were identified. The top regulated gene sets were subjected to coexpression analysis using the Spearman correlation test using the complete linkage algorithm implemented in Genespring 12.0. Only differentially regulated genes in common between the genotypes were considered for further analysis and interpretation.

#### TRIM mutant analysis

The TRIM lines used are described in Table S8. Root morphology was assessed as described above in ‘Root phenotyping’. Genotyping was performed to identify insertion homozygous, heterozygous or WT plants. Roots were imaged, and fixed and root parameters were analyzed using the Winrhizo program.

#### OsNAM_12.1_ constructs and plant transformation

The coding sequence of *OsNAM*_*12.1*_ was amplified from Way Rarem and Vandana total RNA using an RT-PCR system (Promega) according to the manufacturer’s protocol and cloned initially in Topo cloning vector (Zero Blunt^®^ TOPO^®^ PCR, Invitrogen) using gene specific primer pair (NAM101-119F/NAM891-909R). After sequence confirmation, *OsNAM*_*12.1*_ gene was cloned into the binary vector IRS 537, derivative of pCambia 1300 vectors as a *BamHI* and *KpnI* fragment. The construct was confirmed by restriction digestion and sequencing and transformed into *Agrobacterium tumefaciens* strain *LBA* 4404 using freeze-thaw the method^80^. The *Agrobacterium*-mediated plant transformation protocols used were as described previously^81^.

## Additional Information

**How to cite this article**: Dixit, S. *et al.* Action of multiple intra-QTL genes concerted around a co-localized transcription factor underpins a large effect QTL. *Sci. Rep.* **5**, 15183; doi: 10.1038/srep15183 (2015).

## Supplementary Material

Supplementary Information

Supplementary Table 4
