# Supplementary material for: Action of multiple intra-QTL genes concerted around a co-localized transcription factor underpins a large effect QTL
Source: Sci Rep. 2015 Oct 28;5:15183. doi: 10.1038/srep15183 (PMC4623671; doi:10.1038/srep15183)
Supplement: Supplementary Information [file srep15183-s1.pdf]

Action of multiple intra-QTL genes concerted around a co-localized transcription factor underpins a large effect QTL

Shalabh Dixit[1], Akshaya Kumar Biswal[1], Aye Min[1], Amelia Henry[1], Rowena H. Oane[1], Manish L Raorane[1] , Toshisangba Longkumer[1], Isaiah M Pabuayon[1], Sumanth K Mutte[1], Adithi R Vardarajan[1], Berta Miro[1], Ganesan Govindan[1], Blesilda Albano-Enriquez[1] Mandy Pueffeld[2], Nese Sreenivasulu[1,2], Inez Slamet-Loedin[1], Kalaipandian Sundarvelpandian[3], Yuan-Ching Tsai[3], Saurabh Raghuvanshi[4], Yue-Ie C. Hsing[3], Arvind Kumar[1], Ajay Kohli[1]*.

[1]*Plant Breeding, Genetics and Biotechnology Division, International Rice Research Institute, DAPO 7777, Metro Manila-1226, Philippines*

[2]*Leibniz Institute of Plant Genetics and Crop Plant Research (IPK), Corrensstrasse 03, 06466 Gatersleben, Germany.*

[3]*Institute of Plant and Microbial Biology, Academia Sinica, 128 Sec. 2, Academia Road, Nankang, Taipei 11529, Taiwan*

[4]*Department of Plant Molecular Biology, University of Delhi South Campus, New Delhi, 110021, India*

***Correspondence:** a.kohli@irri.org

Keywords: **A**biotic stress, rice, drought, breeding, complex-trait, yield, food security, large-effect QTL, NAC, panicle, root, spikelet, transcription-factor, transcriptome, transgenic.

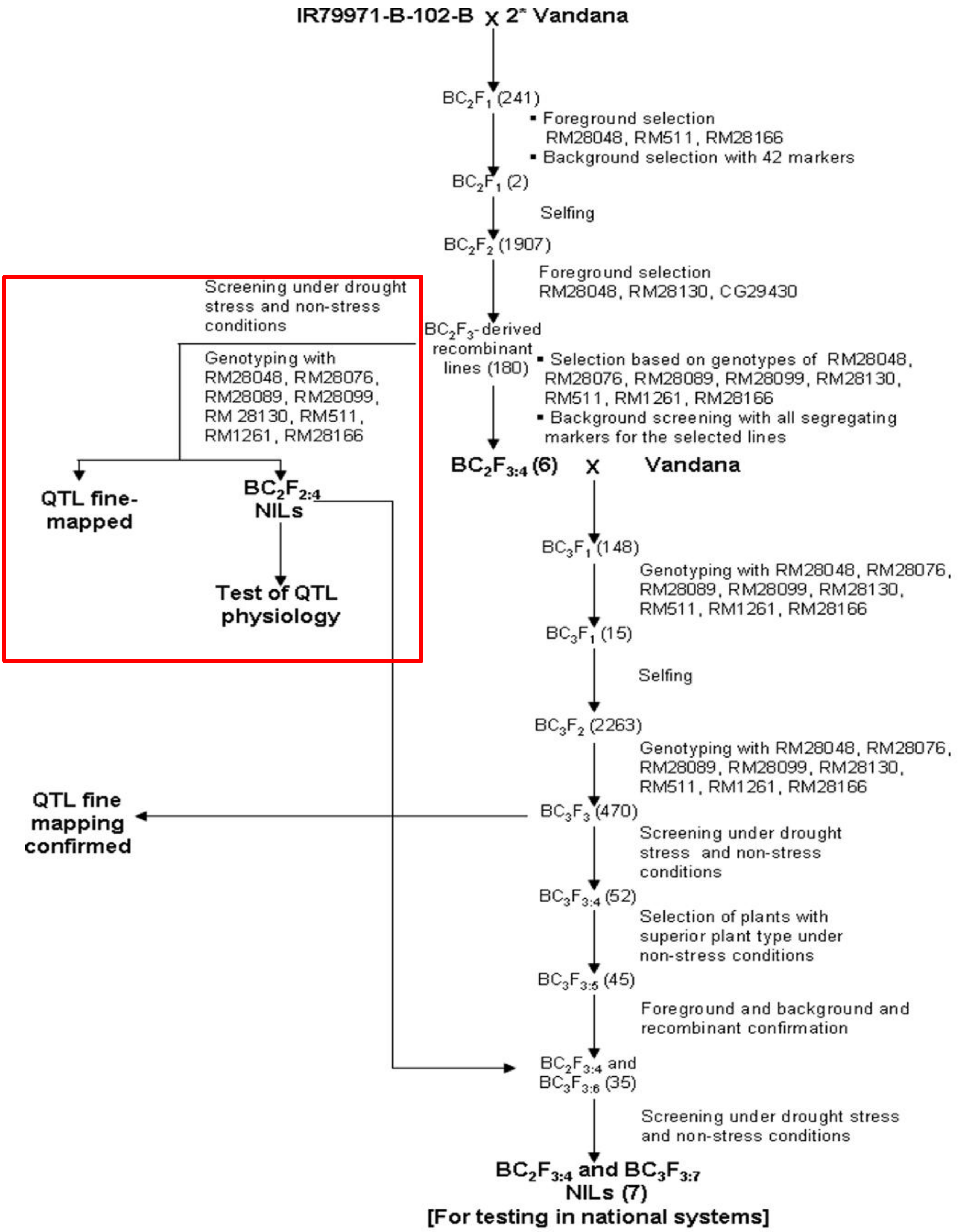

Figure S1: Marker assisted backcrossing (MAB) scheme. MAB scheme used to develop backcross (BC) populations for fine mapping $qDTY_{12.1}$ and development and screening of NILs of recipient parent Vandana. Rice microsatellite (RM) markers which are simple sequence repeats (SSR) were used for genotyping. BC (followed by the number as subscript) refers to the backcross generation; F (followed by the number as subscript) refers to the filial generation developed through selfing after the backcross. RM numbers indicate SSR marker identity.

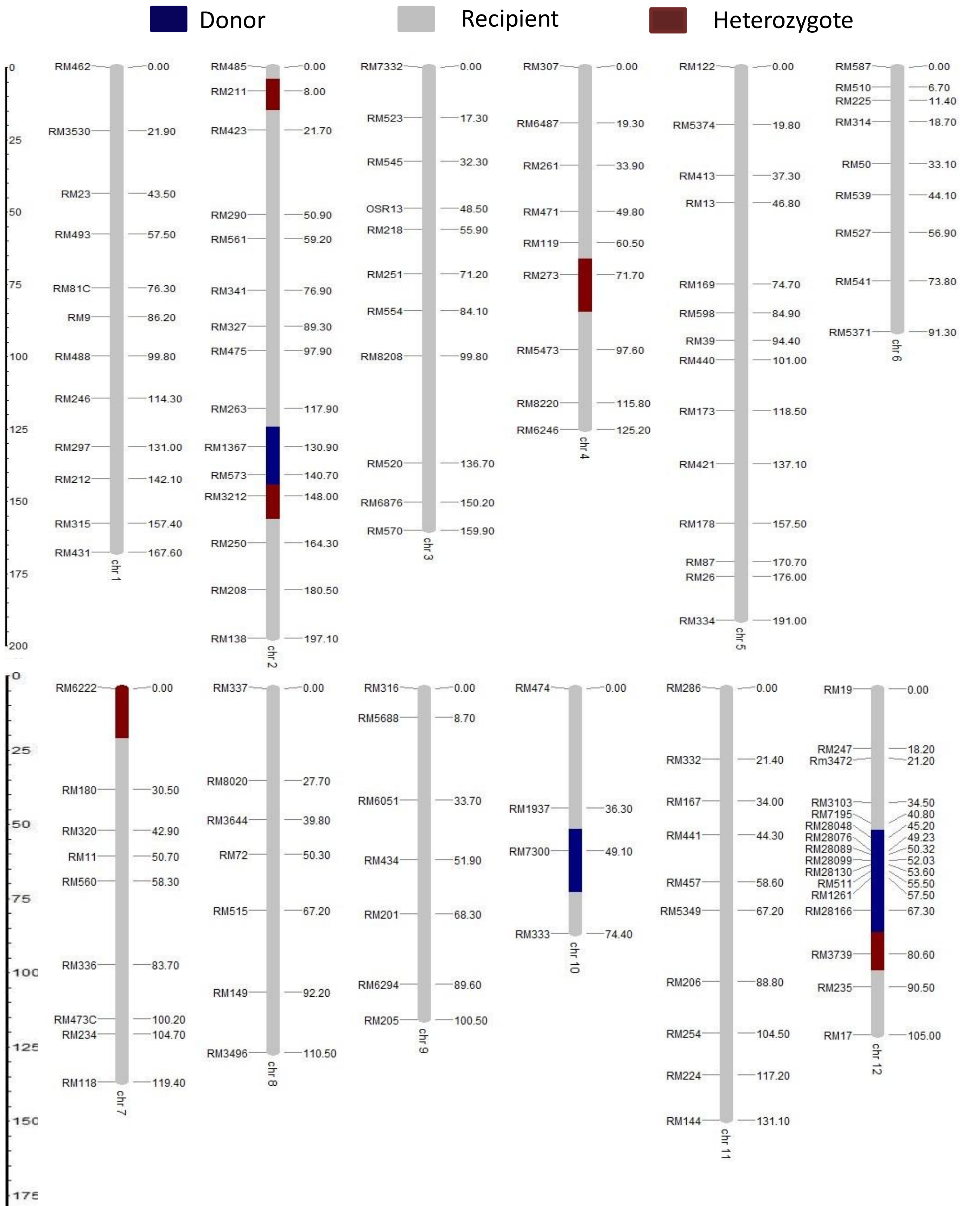

Figure S2: Vandana genome recovery in NIL 481-B. Graphical genotype of NIL 481-B showing recipient genome recovery in the background and length of QTL region transferred in chromosome 12. Blue, Gray and red color on the marker loci represent presence of Vandana (recipient) Way Rarem (donor) and heterozygote allele respectively.

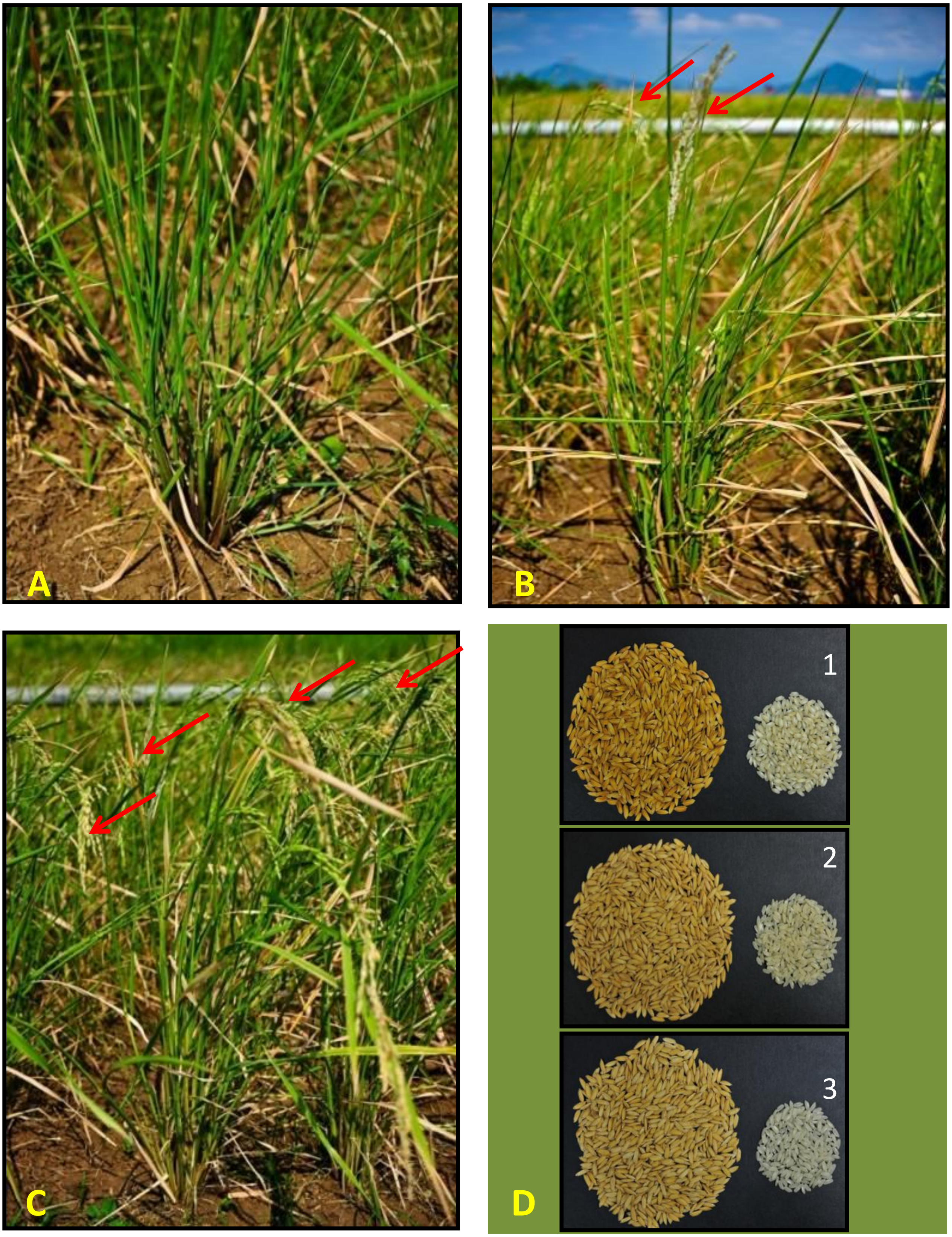

Figure S3: NIL performance under drought. Differences in stress tolerance, plant type and yield under reproductive stage drought in field trial. The donor Way Rarem **(A)** showed less biomass and panicles with filled grains (indicated by red arrows) than the recipient Vandana **(B)** and NIL 481-B **(C).** Grain type of Way Rarem (1) was different while it was highly similar between Vandana (2) and 481-B (3) in bulk harvest (**D**). The grain measurements (in Table S1) and the picture were taken once the NILs were fixed and showed no further within line segregation. The authors acknowledge the 'Communications and Publications Section' (CPS) of the International Rice research Institute for the images in Figure S3 and for permitting the use of these images in this publication.

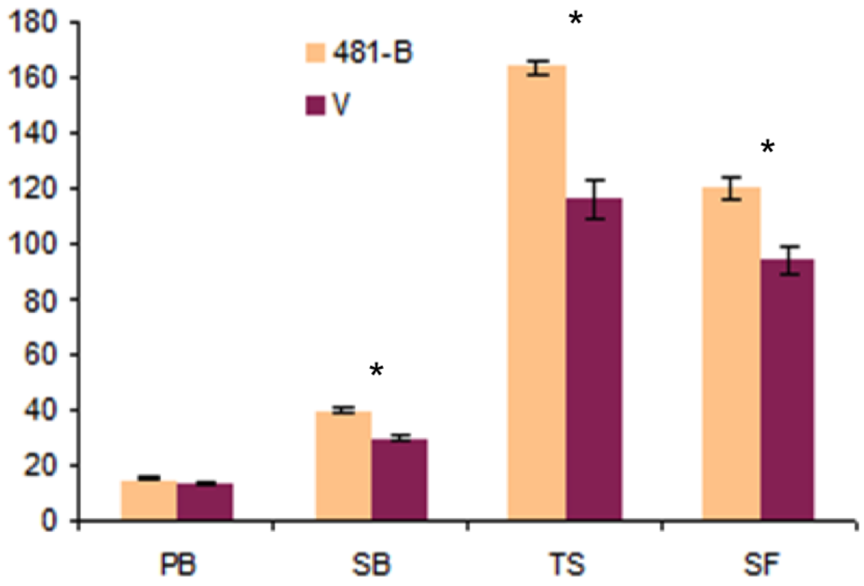

Figure S4: Differences between Vandana and 481-B for panicle branching and yield. Comparison between panicles of Vandana and 481-B revealed similar number of primary branches (PB), but increase in the numbers of secondary branches (SB), total spikelets (TS) and filled spikelets (SF). Three panicles were sampled from 10 plants each under drought in the field. SB had significant increase in 481-B (M=40.6, SD=3.2) when compared to V (M=32.1, SD=2.43); with t(28.4)=1.21e01, p=1.29e-016. Also a significant increase was found in TS in 481-B (M=162, SD=5.3) from V (M=117, SD=9.7); t(27.6)=2.22e01, p=6.37e-26. And also for SF (481-B: M=120, SD4.8; V: M=92.3, SD=6.2); with t(28.7)=1.93e01, and p=4.21e-26.

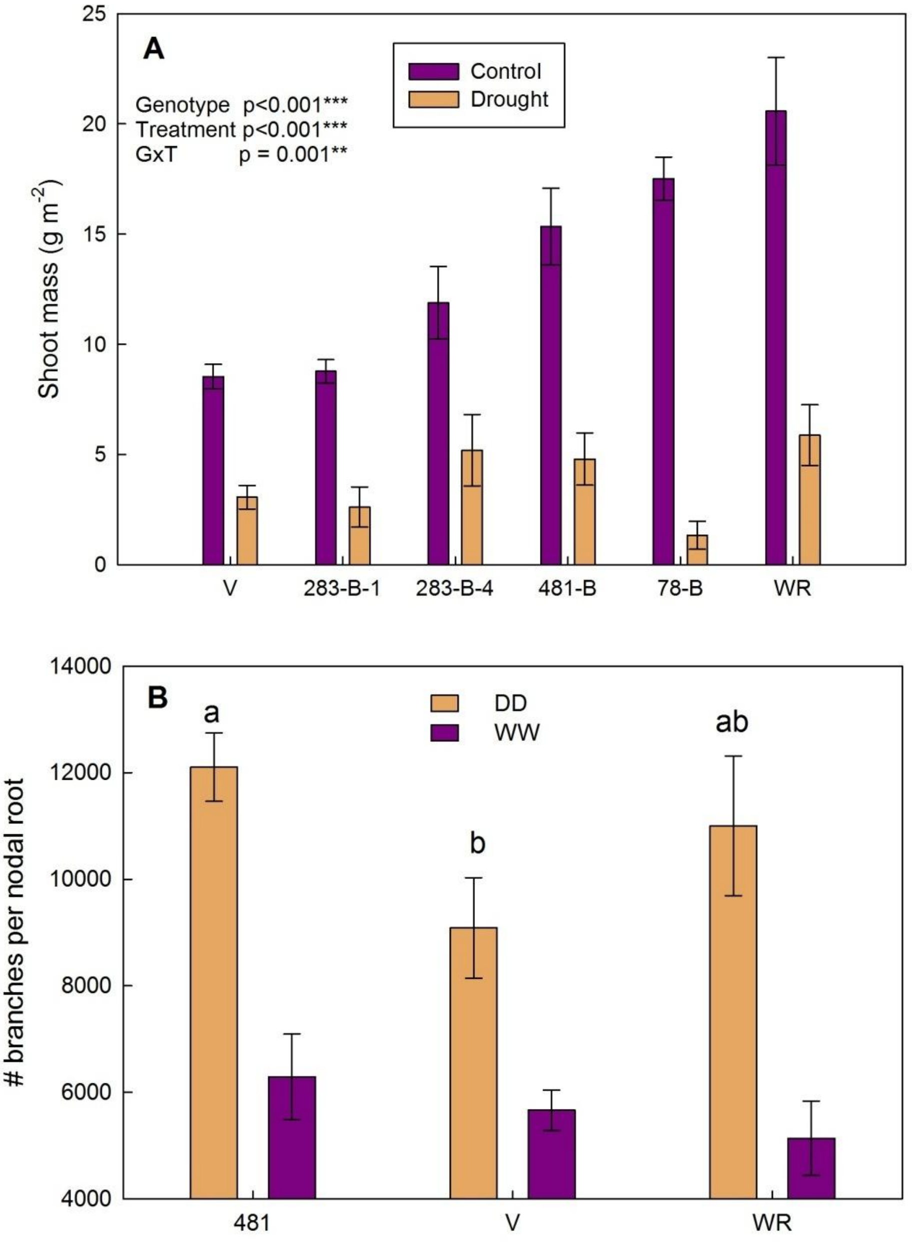

Figure S5. Performance of the NILs seedlings under drought. A) Among 4 NILs assessed, 481-B and 283-B-4 showed improved performance over Vandana at seedling-stage in the field as measured by shoot biomass at 32 DAS (n=4; p<0.001 in the well-watered treatment; p=0.09 in the drought stress treatment). **B)** 481-B showed more root branching than Vandana under seedling stage drought in the greenhouse (n=5, p=0.05). Graphs show mean values ± s.e., and letters indicate different significance groups according to the ANOVA and post-hoc test.

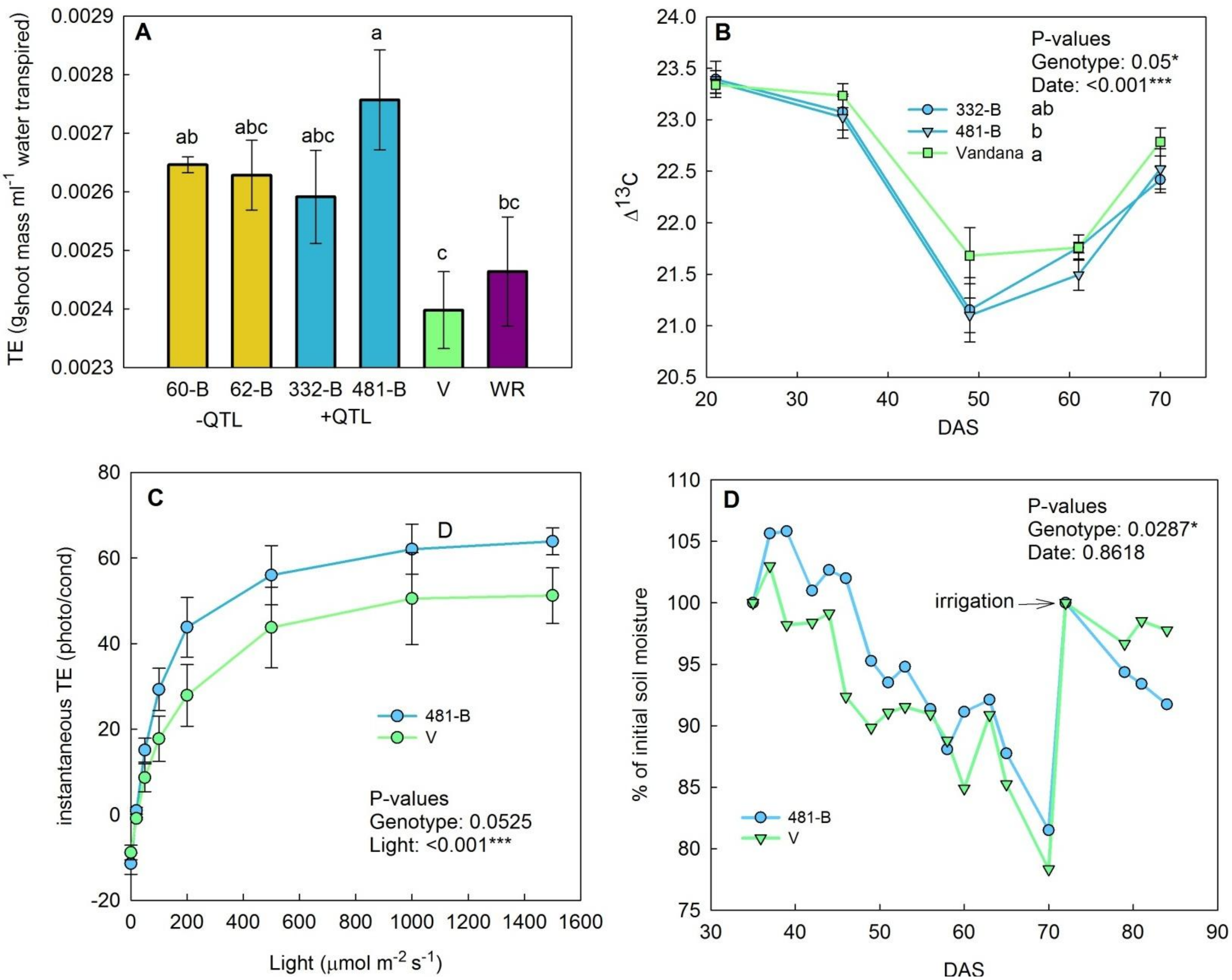

Figure S6. Transpiration efficiency of the NILs. A) Transpiration efficiency was measured in two –QTL (60-B; 62-B) and two +QTL (332-B; 481-B) lines in comparison to Vandana and WayRarem gravimetrically in a greenhouse seedling-stage study (n=5, p=0.04). 481-B was most efficient. **B)** The two QTL+ lines were then compared to Vandana for transpiration efficiency using carbon isotope discrimination in the youngest leaves sampled every 2 weeks during the drought stress period in a field study (n=4, p=0.05). 481-B again exhibited highest transpiration efficiency. **C)** Vandana and 481-B were then directly compared for instantaneous transpiration efficiency by gas exchange (photosynthesis rate/stomatal conductance (n=3, p=0.05) and **D)** by soil moisture measurements in which 481-B showed more conservative water uptake patterns than Vandana during vegetative stage and higher water uptake during reproductive stage at a 40 cm depth (n=3, p=0.0287). Graphs show mean values ± s.e., and letters indicate different significance groups according to the ANOVA and post-hoc test. The drought response of $qDTY_{12.1}$ reflects drought-induced transpiration efficiency coupled with greater water uptake during reproductive stage drought, most likely due to increased lateral root growth.

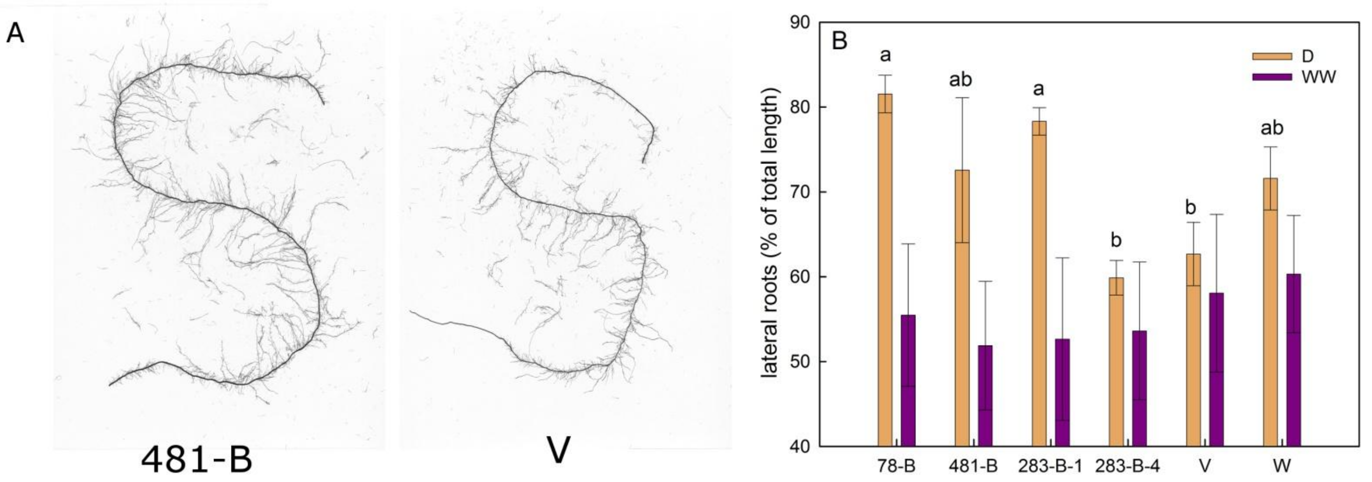

Figure S7. Root branching under drought. A) Under drought more root branches were observed in 481-B compared to Vandana. **B)** Quantitative measurements of root branching in field drought experiments also showed more branches in 481-B (soil depth = 45-60 cm; n=3, p=0.035). The graph shows mean values ± s.e., and letters indicate different significance groups according to the ANOVA and post-hoc test.

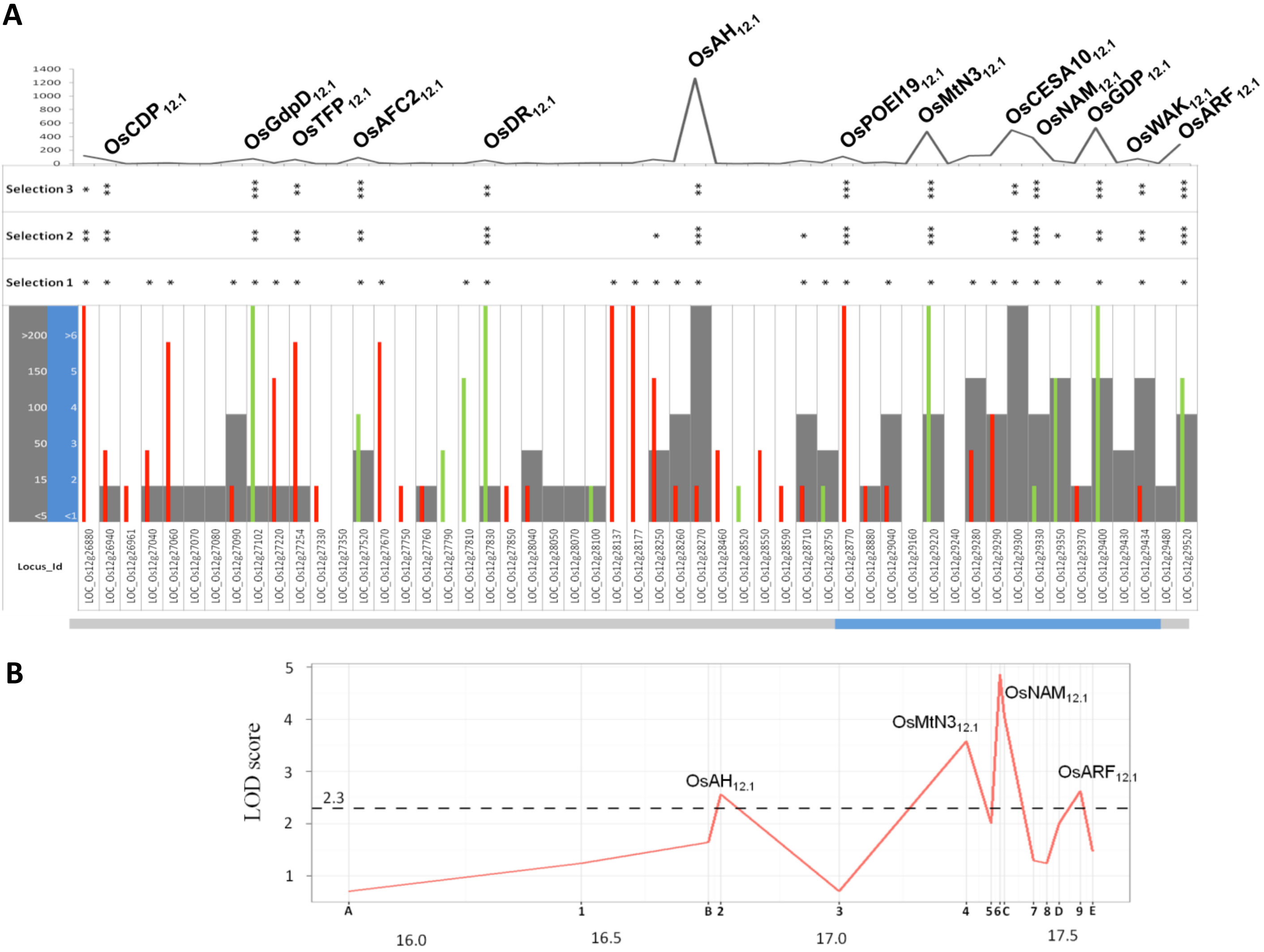

Figure S8. **A) Schematic representation of the selection criterion for gene-based high density markers and candidate genes in *qDTY12.1*.** Candidate genes within the QTL were selected by 3 criteria. First criteria represented by grey barplot was based on the number of SNPs between Vandana and Way Rarem the genes (promoter + coding region). Genevestigator-mediated differential gene expression under drought shows upregulation (green bars) and down-regulation (red bars). Legend for the SNP number is in the left column in gray and for the over-expression/down-regulation fold change in blue. Thirty genes selected based on high number of SNPs and/or high fold change values are marked with 1 star. The second criteria was based on gene co-expression networks assessed from the RiceFREND database for links with other genes relevant to drought tolerance, root or panicle development and water use efficiency as confirmed from peer reviewed literature. A direct association in first hierarchy with any of the three traits was given 3 stars and a secondary relationship was given 2 stars. The last criteria was based on the presence of drought responsive promoter *cis*-regulatory elements, protein domains and protein partners as assessed from PlantCARE/PLACE, Pfam/PROSITE and STRING databases respectively. A gene with 3 or more drought-responsive promoter *cis*-elements and a drought-related protein domain was marked with 3 stars, a gene with only drought-related CIS-elements or drought-related protein domain was marked with 2 stars, and the rest were marked with 1 star (weak relationship) or no star (no relationship). The line plot graphically represents the results of all criteria accounted together. Thirteen putative markers/candidate genes were finally, shortlisted (Figure 3A for full names of the genes). Nine genes ($OsGDP_{12.1}$, $OsAmH_{12.1}$, $OsPOEI19_{12.1}$ $OsMtN3_{12.1}$, $OsCesA10_{12.1}$, $OsNAM_{12.1}$, $OsGRAM_{12.1}$, $OsWAKL3_{12.1}$ and $OsARF_{12.1}$) were selected as colinear markers for high-density mapping. Finally, the hotspot for candidate genes is represented at the bottom of the figure (blue line) over a grey line representing the QTL region. Incidentally maximum SNPs/indels and putative candidate genes occur in this blue region which denotes the original LOD score peak of Bernier et al (2007). **B)** Fine mapping results and identification of sub-QTLs using Bayesian MCMC rqtl package from R software (http://cran.r-project.org/web/packages/qtl/index.html). The fine mapping of the QTL was carried out by using five SSR markers RM28099, RM28130, RM511, RM1261 and RM28166 represented as alphabets and the nine colinear gene markers represented as numerals 1-9 respectively on the x-axis. The LOD threshold was detected at 2.3 and 4 QTLs were identified above the threshold.

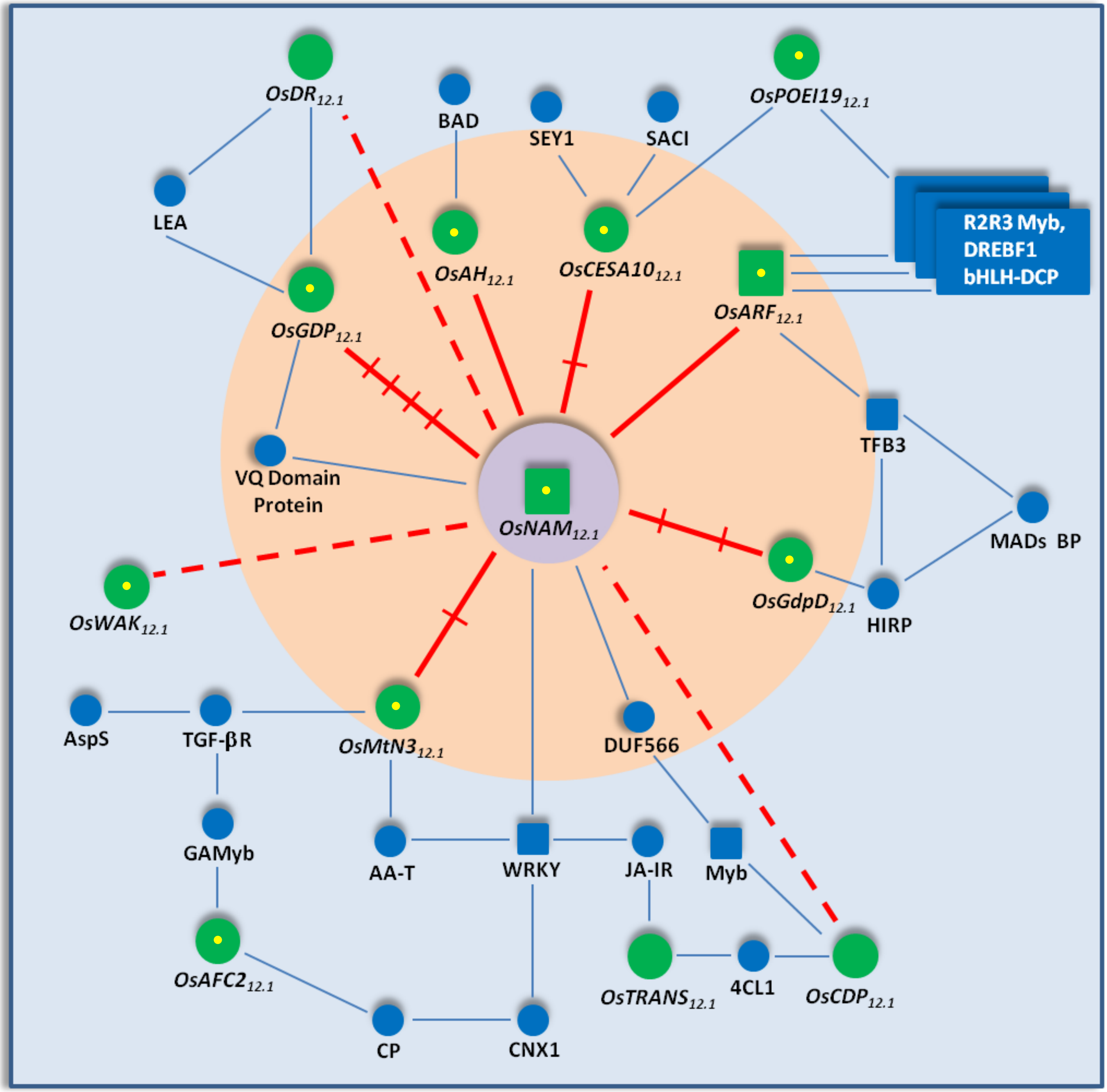

Figure S9. Schematic representation of the possible functional interlinks between the 13 putative candidate genes. Green motifs represent the *qDTY*$_{12.1}$ genes and blue other genomic genes. Boxes are transcription factors. Genes in the beige circle are linked to the central *OsNAM*$_{12.1}$ through solid red lines if validated through EMSA, broken red lines represent transcriptomic evidence of regulation in the transgenic lines overexpressing *OsNAM*$_{12.1}$. Most such links were confirmed at the RiceFrend coexpression database, which was also the basis, along with the STRING database, for the connections depicted by the blue lines. Of the 13 'green' genes, 10 containing the central yellow dot were picked for further analysis based on either a direct link to each other through *OsNAM*$_{12.1}$ or direct links to strongly inducible drought responsive transcription factors. The OsWAK12.1 was an exception and was chosen based on strong evidence from literature on its function relevant to stress tolerance. Details of the genes are shown in the Table S2. Putative interactions between the co-localized genes suggested *qDTY*$_{12.1}$ may be functional through a multi-gene cluster. Gene-clusters of a single GO-term are known for gene families[1], biosynthetic pathways[2] or traits[3, 4]. QTLs functional through multiple genes, each of a separate GO-term have not been validated.

References: **1)** Li et al. (2007) Identification of Six New Box C/D snoRNA Gene Clusters from Rice. IUBMB Life, 59: 664 – 674. **2)** Shimura et al. (2007) Identification of a Biosynthetic Gene Cluster in Rice for Momilactones. JBC, 282: 34013-34018. **3)** He et al. (2006) Haplotype variation in structure and expression of a gene cluster associated with a quantitative trait locus for improved yield in rice. Genome Research 16: 618-626. **4)** Yang et al. (2012) A Killer-Protector System Regulates Both Hybrid Sterility and Segregation Distortion in Rice. Science 337: 1336-1340.

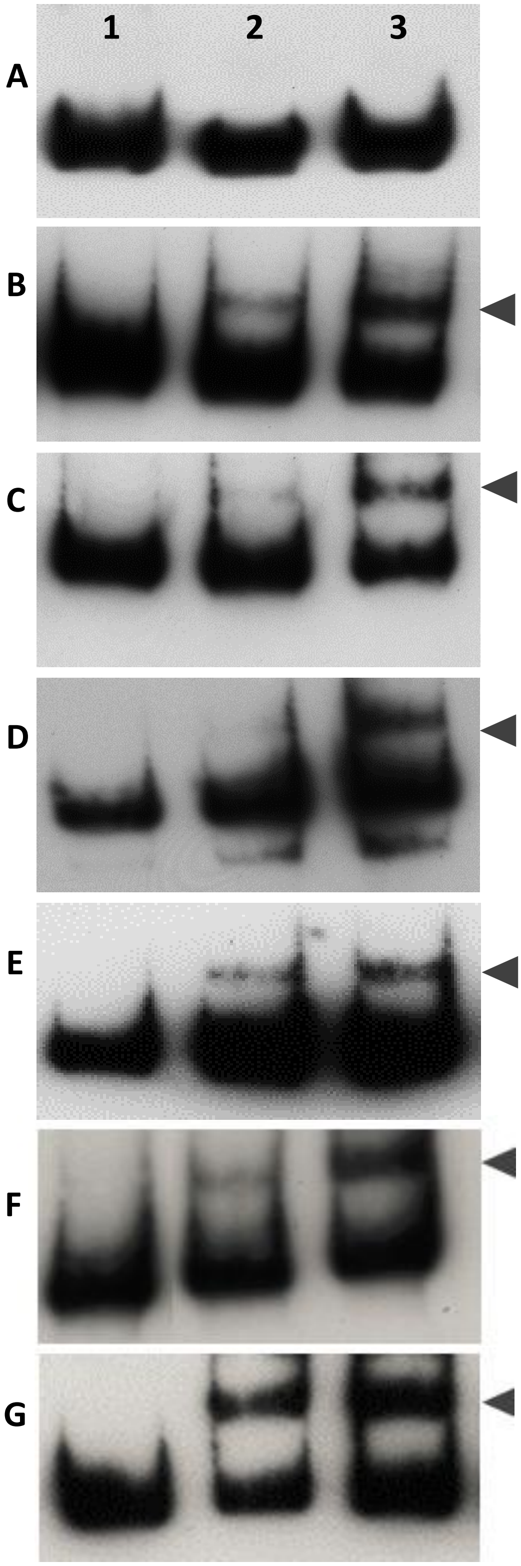

Figure S10. EMSA-mediated assay for gene promoter:*OsNAM12.1* binding. Lane 1. Free labelled probe (promoter fragment). **Lane 2.** r-*OsNAM* $_{12.1}$ + labelled probe + specific competitor (unlabelled probe fragment)**. Lane 3.** r-*OsNAM*$_{12.1}$ + labelled probe. Alphabets A to F respectively represent *OsGdpD12.1* fragment 4 as the negative control, *OsGDP*$_{12.1}$; *OsCESA*$_{12.1}$; *OsARF*$_{12.1}$; *OsMtN3*$_{12.1}$; *OsGdpD*$_{12.1}$ fragment 5 and *OsAH*$_{12.1}$ respectively. Arrow marks indicate the band shift due to a complex between the specific promoter and r-*OsNAM*$_{12.1}$. In each case, except the negative control, promoter:protein binding increases in lane 3 suggesting specific binding of *OsNAM*$_{12.1}$. The promoter fragments assessed were obtained from the donating parent Way Rarem

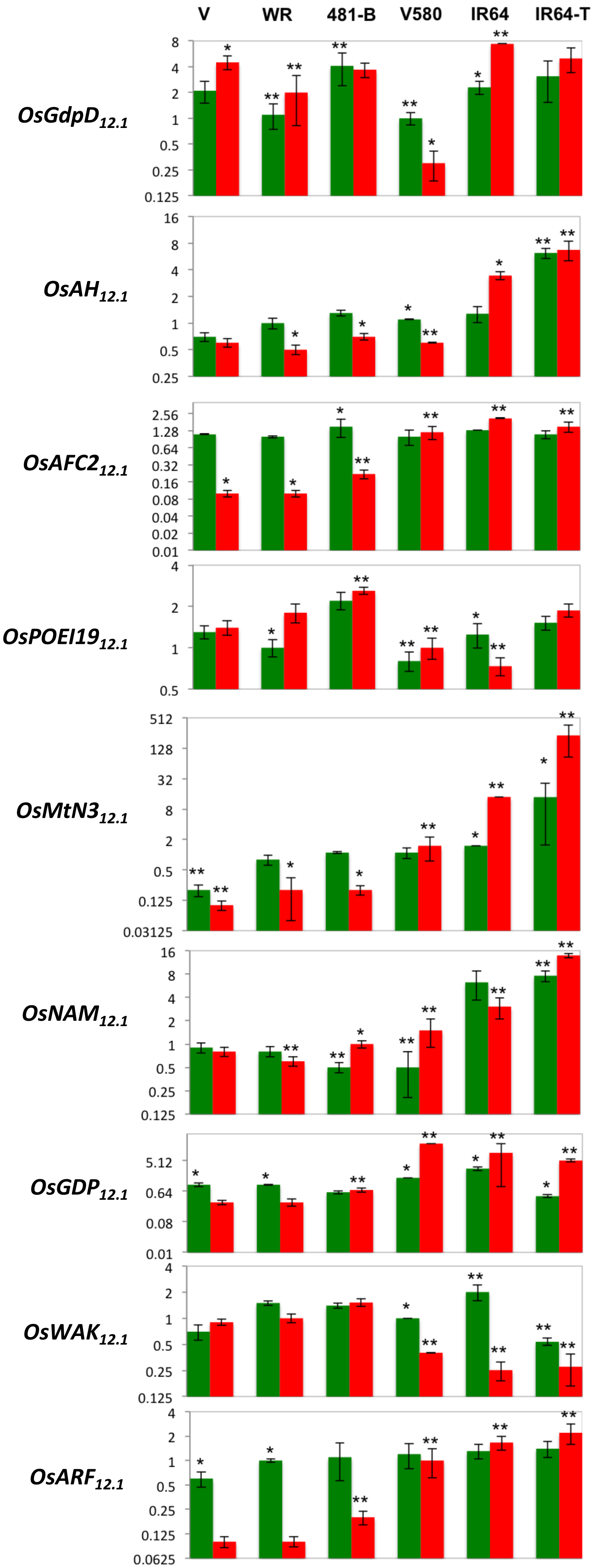

Figure S11. Candidate gene expression under drought.

Bar graphs represent expression of the 9 candidate genes assessed by qRTPCR in 6 lines in the roots under well watered (green bars) and drought (red bars) conditions at the heading stage. The candidate genes are listed vertically on the left. Genotypes listed horizontally at the top are the parental Vandana (V) and Way Rarem (WR), NIL (481-B), transgenic Vandana overexpressing the Way Rarem $OsNAM_{12.1}$ (V580), IR64 wild type (IR64) and IR64 overexpressing the Way Rarem $OsNAM_{12.1}$ (IR64-T). Gene expression values were normalized against cyclophilin as an internal reference gene. Expression is represented in the y axis as a log2 scale. Significantly different expression between well watered and drought conditions is symbolized with * (one star) and significantly different expression between the genotypes is symbolized with ** (2 stars). Significance was assessed with a two-tailed t-test at p=0.005.

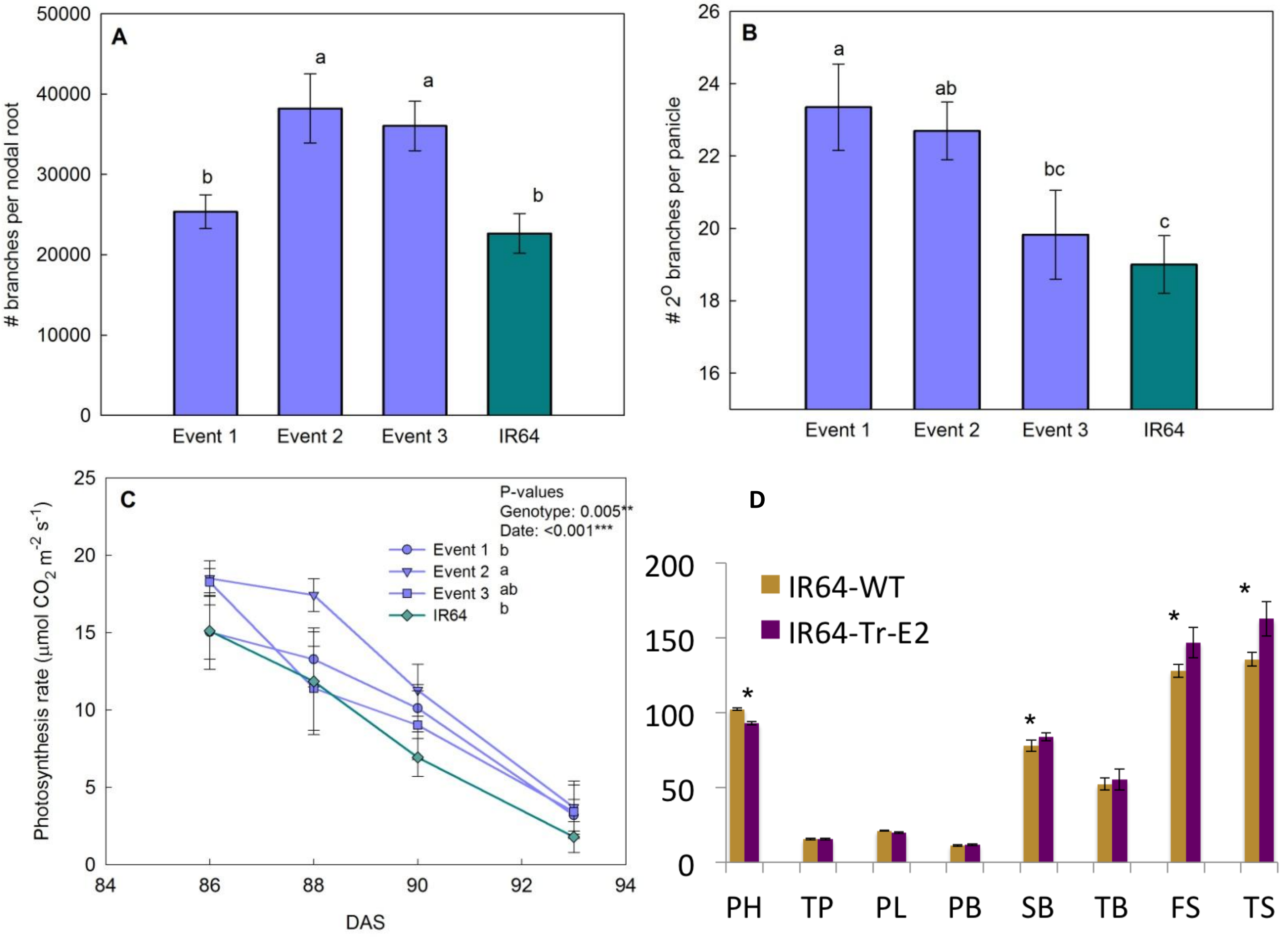

Figure S12. Assessment of the transgenic plants. Three independent transgenic events overexpressing *OsNAM12.1* exhibited **A)** increase in root branching (n=8, p=0.005); **B)** increase in panicle branching (n=10, p=0.012) and **C)** increase in transpiration in the drought stress treatment (n=4, p=0.005). Graphs show mean values ± s.e., and letters indicate different significance groups according to the post-hoc test. Event E2 stood out as potentially useful due to increase in root branching and transpiration. **D)** Due to its favorable characteristics event E2 was compared with the wild type IR64 for panicle traits affecting yield under drought; plant height (PH, in cm); number of tillers per plant (TP); panicle length (PL, in cm); number of primary branches (PB); secondary branches (SB); tertiary branches (TB); fertile spikelets (FS) and total spikelets (TS). Bar graph plotted for each trait showing mean (M) and standard error of 12 and 9 measurements for the wild type (IR64-WT) and transgenic plant (IR64-Tr-E2), respectively. PH was significantly decreased while SB, FS and TS were significantly increased. These changes led to marginally higher 100-grain weight in IR64-Tr-E2 than IR64-WT (for IR64-Tr-E2: M=2.6 and SD=0.13, for WT: M=2.4 and SD=0.18; t(18.9)=-3.035, p=0.0068).

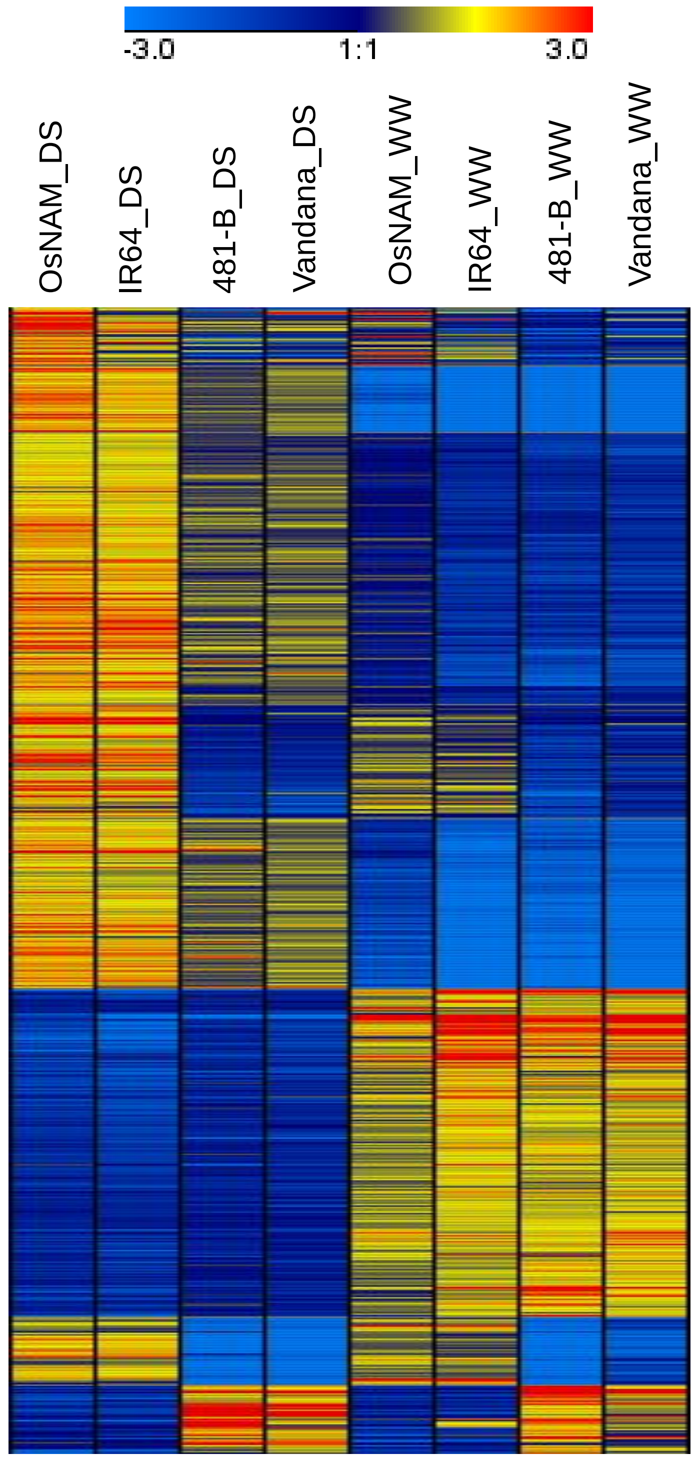

Figure S13. Heatmap analysis of microarray data. Temporal expression profiles of differentially expressed genes in roots between *OsNAM*$_{12.1}$ transgenic line in IR64 background, IR64 (wild type), 481-B near isogenic line and Vandana under drought stress (DS) and well watered (WW). By using K-mean and SOM clustering methods, four cluster groups were defined based on temporal expression patterns. Note that cluster group 1 genes are mostly expressed under DS. On the other hand, cluster group 2 transcripts are preferentially expressed under WW but down-regulated under DS across all genotypes. The cluster group 3 transcripts regulated under WW and DS in OsNAM and IR64 and cluster group 4 transcripts are expressed under WW and DS in 481-B and Vandana lines. Expression values are given in logarithmically scaled (base 2) signal intensities: red, high expression; yellow, moderate expression; blue, low expression. Horizontal rows represent gene expression patterns.

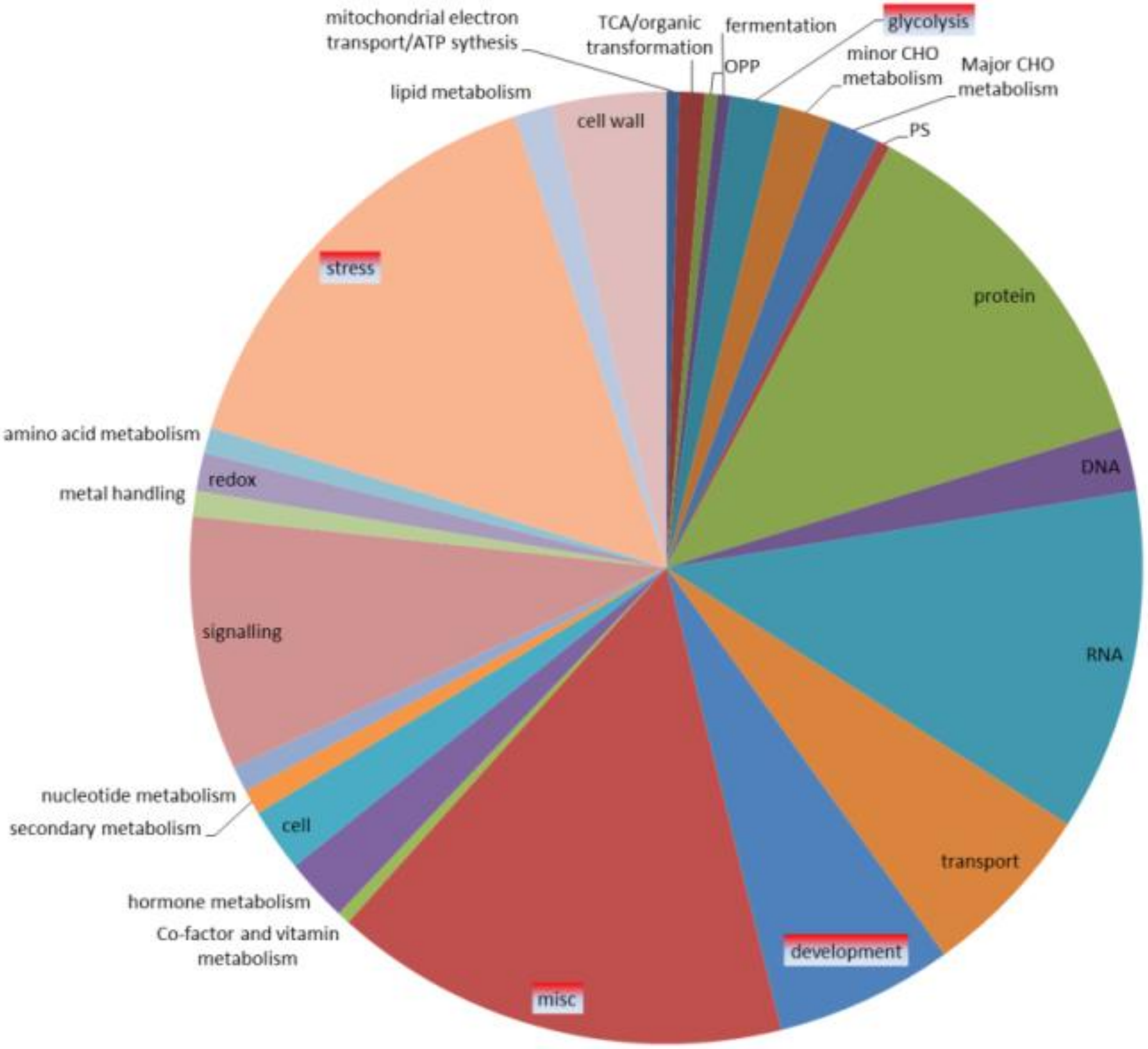

Figure S13 B) Functional categories of the genes upregulated under drought as identified from transcriptome analysis of the roots

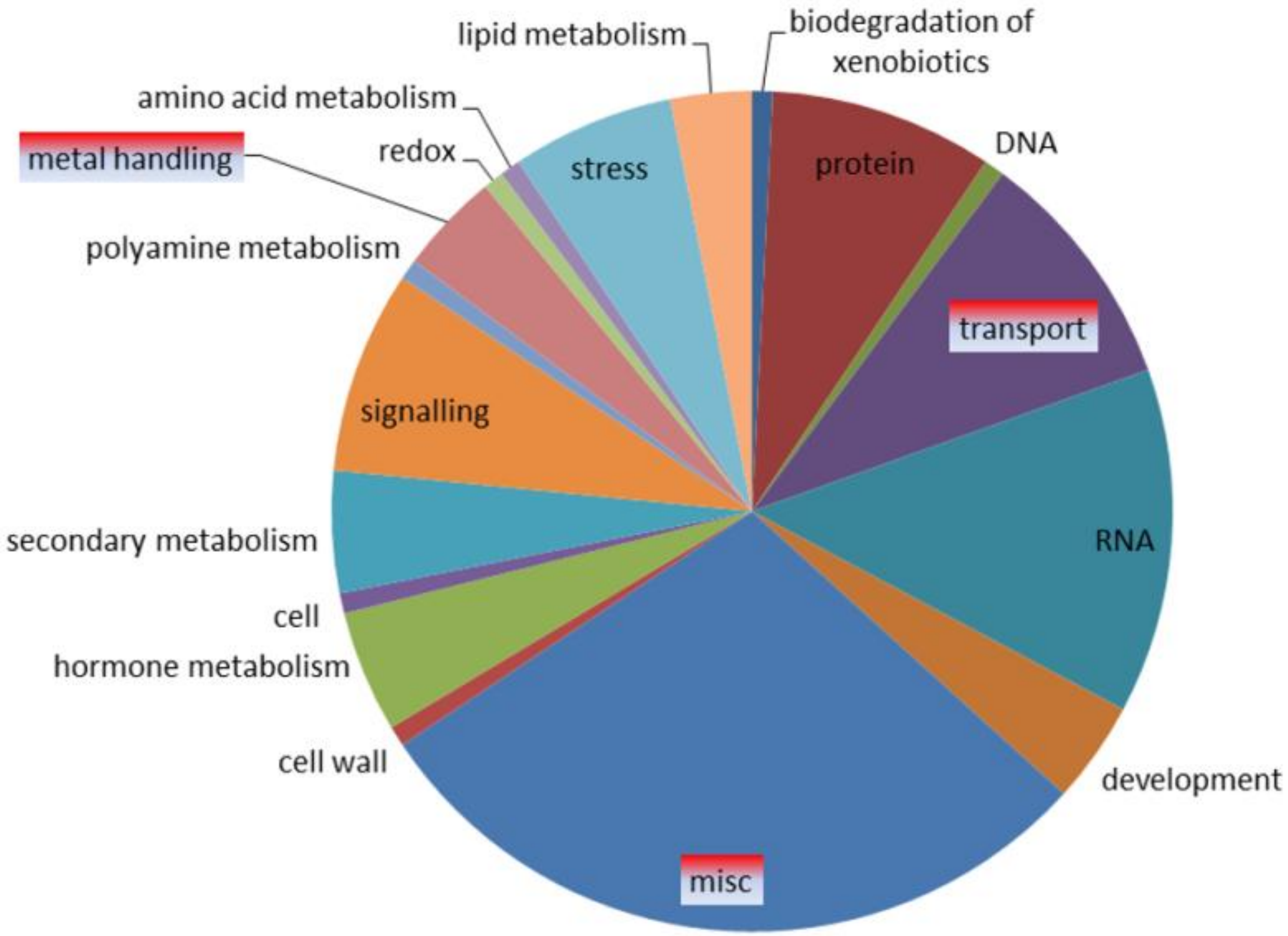

Figure S13 C) Functional categories of the genes down-regulated under drought as identified from transcriptome analysis of the roots

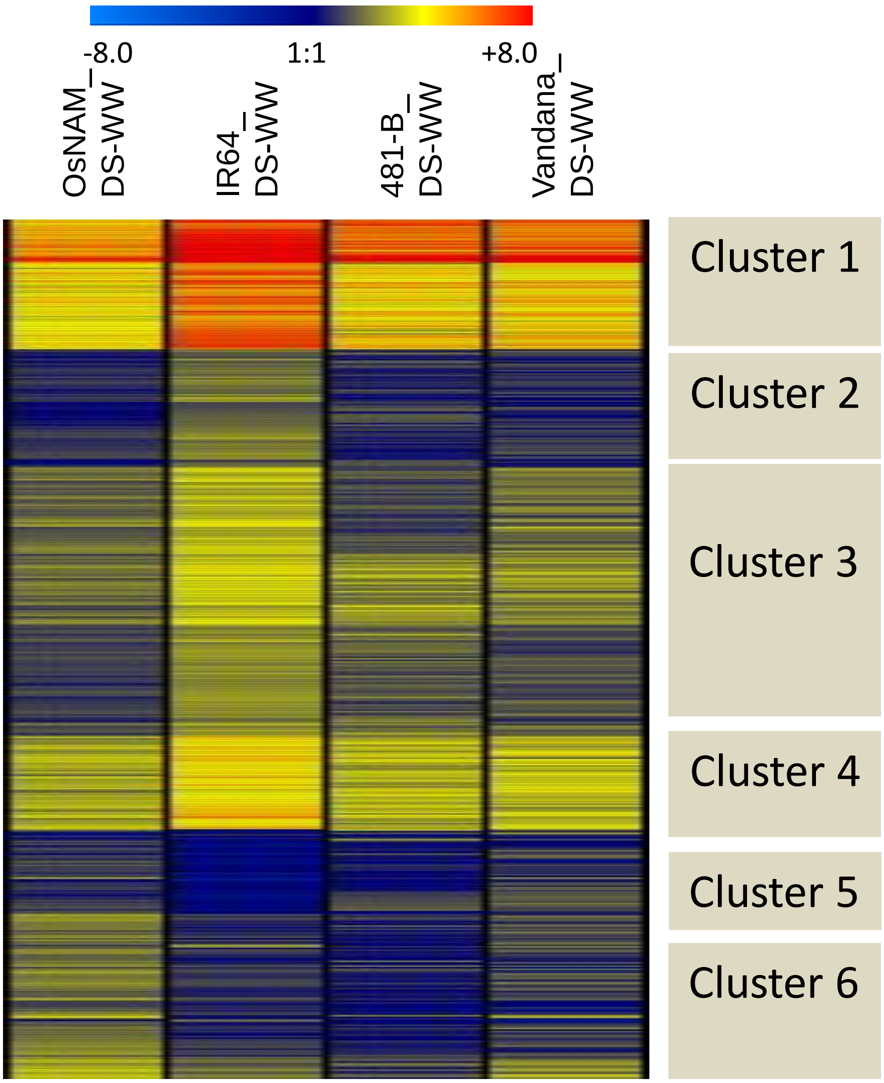

Figure S14. Heatmap analysis of microarray data. Expression profiles of rice genes responsive to drought depicted as heat-map. Expression ratios (drought stressed vs well watered) are colour-coded: Red >8 fold up-regulated under DS; blue >8 fold up-regulated under WW. Genes were grouped into 6 clusters based on hierarchical clustering using the average linkage method. Each gene is represented as horizontal row and the vertical columns represent tissue samples (OsNAM transgenic line in IR64 background, IR64, 481-Bnearisogenic line, Vandana)

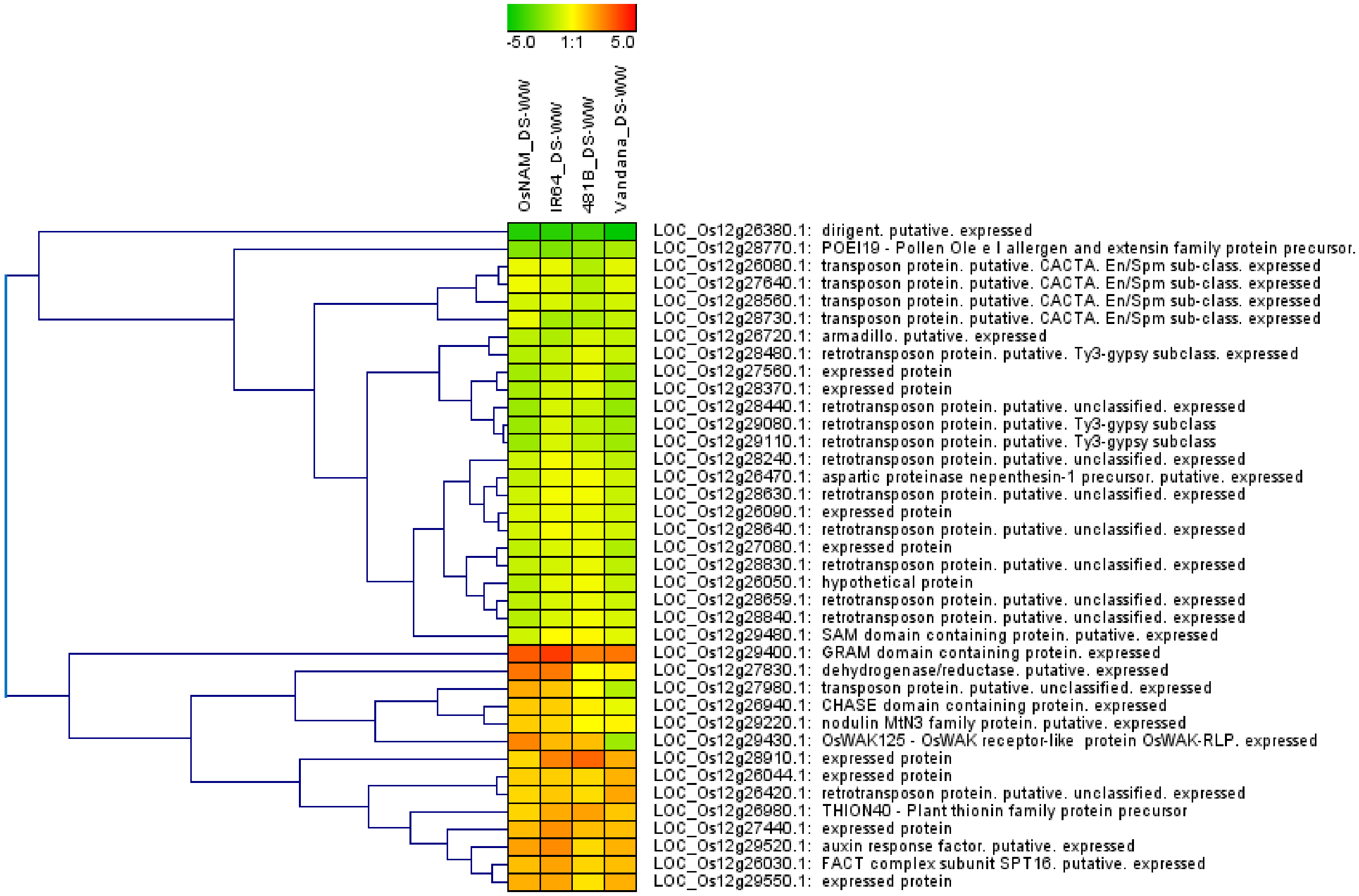

Figure S15. Dendrogram of microarray data for genes within q*DTY*$_{12.1}$. The fold differences calculated between drought stressed (DS) vs well watered (WW) root samples in OsNAM, IR64, 481-B near isogenic line and Vandana lines shown as heat map. Hierarchical clustering of 38 differentially expressed genes of *qDTY*$_{12.1}$ led to two major clusters. Green or red colors indicate differentially down and up-regulated genes under DS, respectively. Genes were grouped into 2 clusters based on hierarchical clustering using the average linkage method.

A

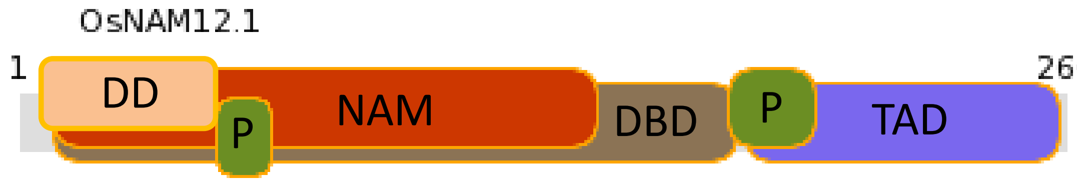

B

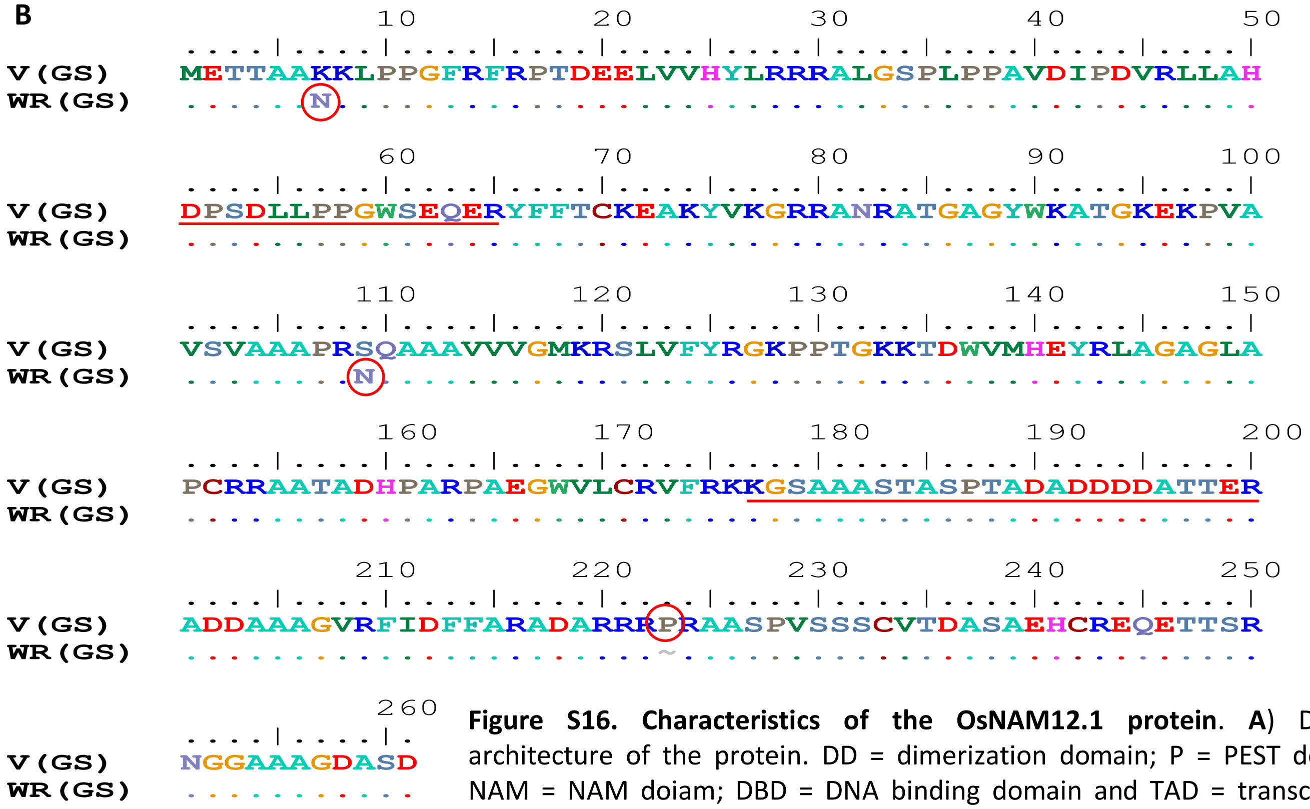

C

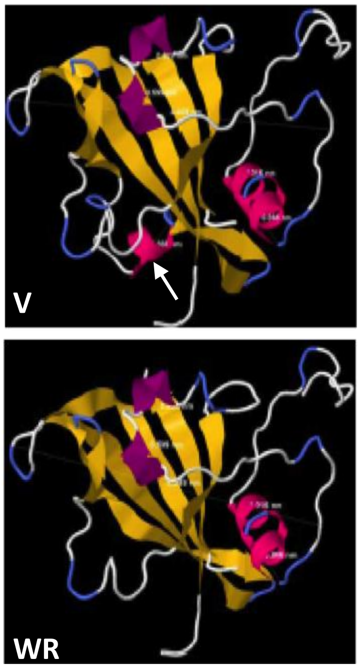

Figure S16. Characteristics of the OsNAM12.1 protein. A) Domain architecture of the protein. DD = dimerization domain; P = PEST domain; NAM = NAM doiam; DBD = DNA binding domain and TAD = transcription activation domain. **B)** Protein sequence comparison between Vandana and WayRarem. Changed AAs encircled. PEST sequence underlined **C)** Predicted effect of AA changes on protein structure indicated by the white arrow.

Among the 114 rice NAM proteins, *OsNAM*$_{12.1}$ was one of only eight predicted to contain two PEST motifs, a protein degradation signal present in the negative regulatory domain of DREB2A. It was one of only five whose PEST motif was predicted to be potentially ubiquitinated and SUMOylated and prone to intrinsically disordered protein structure. These modifications actively regulate the fate of the PEST motif and hence the activity of the motif-containing protein[1]. Also, among the 268 rice and Arabidopsis NAM/NAC genes, *OsNAM*$_{12.1}$ phylogenetically belongs to the ONAC1 clade of eight members[2], none of which are characterized. Thus, effects of *OsNAM*$_{12.1}$ on roots, and on the yet to be analyzed panicle branching, transpiration efficiency and yield under drought, might be due to a unique combination of *OsNAM*$_{12.1}$ *per se*, it's genomic location and it's *cis*- and *trans*-interactions with other genes/proteins.

References: 1) Singh et al. (2006) Intrinsic unstructuredness and abundance of PEST motifs in eukaryotic proteomes. PROTEINS: Structure, Function, and Bioinformatics 62:309–315. **2)** Nuruzzaman et al. (2010) Genome-wide analysis of NAC transcription factor family in rice. Gene 465(1-2):30-44

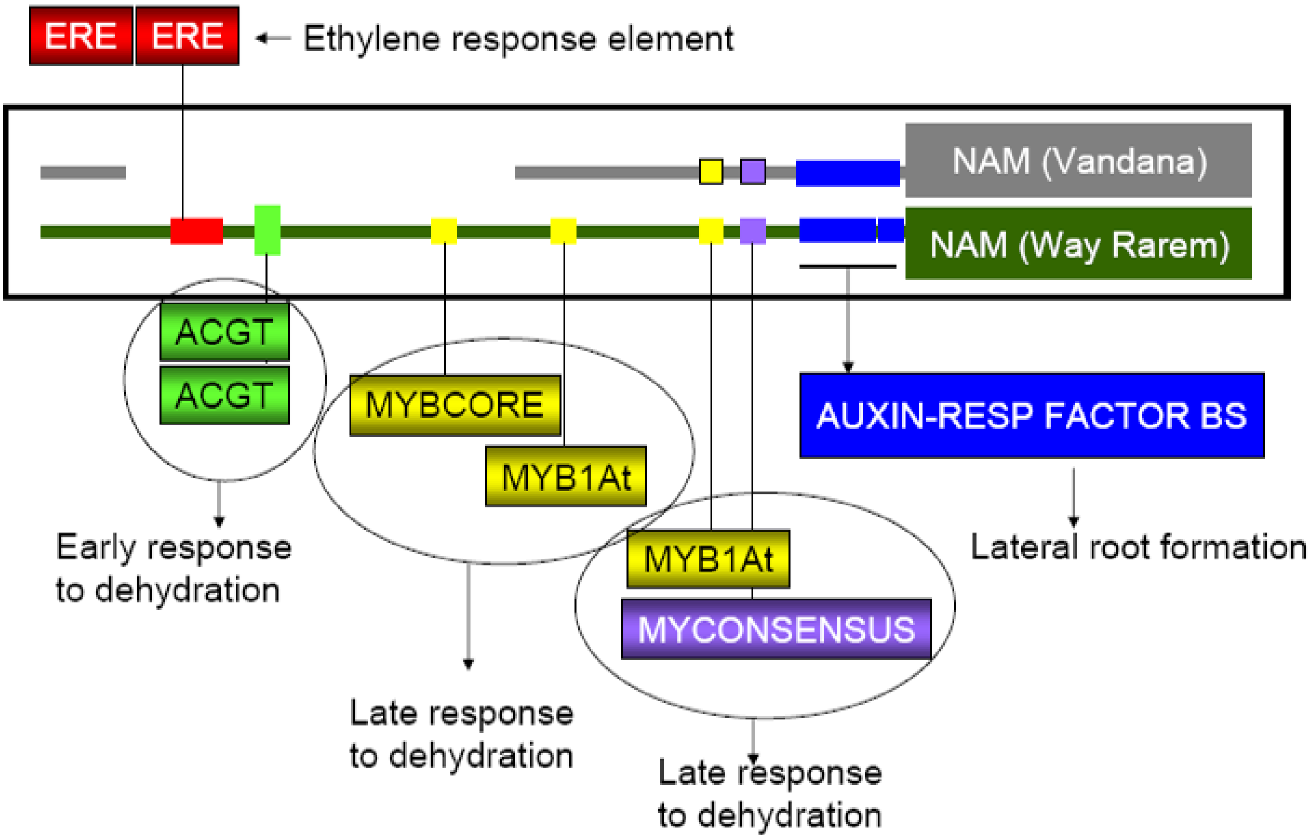

Figure S17. An indel in the promoter region of *OsNAM*$_{12.1}$. Important drought responsive and root development associated *cis*-regulatory elements were missing in the Vandana allele, particularly the ethylene response elements and some elements responsive to dehydration.

Table S1. Field performance and grain characteristics of $BC_2F_{3:4}$ and $BC_3F_{3:7}$ NILs, parents, and drought-tolerant checks in advanced yield trials under upland severe stress and non-stress conditions with respective percentage recovery of Vandana allele in the background (%BG)

		**Upland severe stress**					**Upland non-stress**								
**Genotype**	**Particulars**	**GY**	**DTF**	**PHT**	**BIO**	**HI**	**GY**	**DTF**	**PHT**	**BIO**	**HI**	**GL**	**GW**	**L:W**	**% BG**
IR84984-83-15-481-B	$BC_2F_{3:4}$	693	64	75	4160	0.17	2525	62	79	9584	0.29	0.65	0.25	2.6	93.4
IR84984-83-15-862-B	$BC_2F_{3:4}$	582	66	75	5120	0.28	2037	67	86	11050	0.29	0.64	0.25	2.6	92.6
IR84984-21-19-78-B	$BC_2F_{3:4}$	478	66	74	3333	0.13	2996	65	89	10769	0.31	0.63	0.25	2.5	92.6
IR84984-83-15-110-B	$BC_2F_{3:4}$	475	66	78	3210	0.3	2279	66	89	8539	0.29	0.64	0.24	2.7	92.6
IR90020:22-283-B-1-B	$BC_3F_{3:7}$	604	66	79	4160	0.14	3039	66	85	11610	0.29	0.64	0.23	2.8	93.4
IR90020:22-283-B-4-B	$BC_3F_{3:7}$	515	67	77	4133	0.21	2245	68	84	7579	0.27	0.65	0.26	2.5	95.0
IR90019:17-156-B-2-B	$BC_3F_{3:7}$	323	67	75	4650	0.18	2202	67	87	8539	0.36	0.63	0.25	2.5	95.9
Way Rarem	Donor	0	NF	60	2267	0.01	1660	87	100	NA	NA	0.62	0.28	2.2	-
Vandana	Recipient	27	70	79	2080	0.09	2208	68	91	10498	0.34	0.63	0.24	2.6	-
Apo	Check	0	NF	57	2267	0	2765	80	101	NA	NA	0.65	0.24	2.7	-
UPLRi7	Check	0	NF	55	1290	0	2236	85	91	NA	NA	0.68	0.24	2.8	-
IR74371-54-1-1	Check	0	NF	65	2960	0	2935	75	86	NA	NA	0.6	0.23	2.6	-
Population mean		327	68	76	4150	0.13	2461	67	87	10497	0.31	-	-	-	-
**p value**		<0.0001	0.0005	<0.0001	0.0299	<0.0001	0.011	<0.0001	0.0233	0.002	0.097	-	-	-	-

DTF: days to 50% flowering, PHT: plant height (cm), GY: grain yield (kg ha^{-1}), BIO: shoot biomass (kg ha^{-1}), HI: harvest index GL: grain length, GW: grain width, L:W: grain length-width ratio, NF: no flowering, NA: not available

Table S1 presents the summary of yield, phenology and plant type related traits for selected NILs in field trials conducted under reproductive stage drought stress and non-stress conditions. The table also shows the grain measurements (under non-stress) and percentage background recovery. The experiments were conducted in alpha lattice design with three replicates and included NILs, parents and check varieties. Data on yield, biomass, height, days to flowering and harvest index were recorded and analyzed using mixed model analysis to compute LS means and P values. Genotype, blocks and replication were taken as factor with effects of genotypes taken as fixed effects while those for blocks and replications were taken as random effects. Grain measurements were conducted on non-stress grains harvested from the NILs, parents and checks. Length and width of grains were calculated from observations taken from 10 grains of each line. L:W ratio was calculated based on the mean length and width data.

q*DTY*$_{12.1}$ genes		Other genes		MSU name
LOC_Os12g29300	*OsCESA10*$_{12.1}$	SEY1	LOC_Os12g41170	SEY1 putative
		SACI	LOC_Os11g20384	SacI homology domain containing protein
LOC_Os12g28770	*OsPOLEI19*$_{12.1}$	R2R3	LOC_Os07g43580	MYB family transcription factor
LOC_Os12g29520	*OsARF*$_{12.1}$	Myb	LOC_Os10g30690	MYB family transcription factor, putative,
		DREB1F	LOC_Os01g73770	dehydration-responsive element-binding protein
		bHLH-DCP	LOC_Os01g01870	helix-loop-helix DNA-binding domain CP
		TFB3	LOC_Os11g32110	Auxin response factor
		MADs BP	LOC_Os01g10504	MADS-box family gene with MIKCc type-box
LOC_Os12g27102	*OsGdpD*$_{12.1}$	HIRP	LOC_Os09g19710	hypersensitive-induced response protein,
LOC_Os12g26940	*OsCDP*$_{12.1}$	Myb	LOC_Os04g50770	MYB family transcription factor, putative,
		4CL1	LOC_Os08g14760	4-Coumarate ligase/AMP binding DCP
LOC_Os12g27254	*OsTFP*$_{12.1}$	JA-IR	LOC_Os12g14440	Jacalin - Jasmonate-induced protein, putative
LOC_Os12g29330	*OsNAM*$_{12.1}$	DUF566	LOC_Os10g40620	Domain of unknown function containing protein
		WRKY	LOC_Os01g47560	WRKY16, expressed
LOC_Os12g29400	*OsGDP*$_{12.1}$	VQ DP	LOC_Os05g44270	VQ domain containing protein, putative, expressed
LOC_Os12g27830	*OsDR*$_{12.1}$	LEA	LOC_Os08g23870	late embryogenesis abundant group 1, putative
		CNX1	LOC_Os04g56620	molybdopterin biosynthesis protein CNX1, putative
LOC_Os12g29220	*OsMtN3*$_{12.1}$	AA-T	LOC_Os08g03350	amino acid transporter, putative
		TGF-BR	LOC_Os02g37040	TGF-beta receptor, type I/II extracellular protein
LOC_Os12g27520	*OsAFC2*$_{12.1}$	CP	LOC_Os10g42790	CTP:phosphorylcholine cytidylyltransferase
		GAMyb	LOC_Os03g63020	Gibberellin activated Myb binding kinase
		AspS	LOC_Os03g18130	asparagine synthetase, putative
LOC_Os12g28270	*OsAH*$_{12.1}$	BAD	LOC_Os04g39020	Betaine aldehyde dehydrogenase, putative
LOC_Os12g29434	*OsWAK*$_{12.1}$			

Table S2. Details on the IDs of the genes with network relationship among the *qDTY*$_{12.1}$ genes and other genes. Green font represents q*DTY*$_{12.1}$ genes and blue font their networked gene links. The beige and light blue color overlap with the 'blue' genes imperfectly captures the links depicted in Figure S9. Purple shaded genes are common links between two QTL genes. Nearly all blue genes have functional links to at least one of the traits affected by q*DTY*$_{12.1}$ i.e. drought tolerance, root and panicle branching, water use efficiency and yield. For the q*DTY*$_{12.1}$ genes *CESA*$_{12.1}$ and *OsARF*$_{12.1}$ belong to two classes of genes closely associated with root development[1,2], especially in combination with NAM/NAC TFs[3]. Gram domain proteins (*OsGDP*$_{12.1}$) are ABA responsive proteins[4] and ABA content has a bearing on drought response[5]. *OsAFC2*$_{12.1}$ has 72% identity to the *Arabidopsis* and *Brachypodium* AFC2-LAMMER kinase, which may modulate the transcriptome through alternative splicing, mediated by its known potential to phosphorylate and activate the spliceosome component S/R proteins[6]. *OsWAK*$_{12.1}$ is similar to the WAK receptor-like kinases in the cell wall that are associated with signaling for modification of cell wall integrity during expansion. Silencing of a WAK in rice led to abnormal root growth along with defective anther dehiscence[7]. The *OsPOlEI*$_{12.1}$ has high similarity to an Arabidopsis gene (AT5GO5500) known to be functional in root hair elongation. Other than the result that *OsGdpD*$_{12.1}$ and *OsMtN3*$_{12.1}$ were upregulated under drought, lack of additional information on them at this stage warrants more research, especially on *OsMtN3*$_{12.1}$ because along with *OsNAM*$_{12.1}$ it subtends the major LOD peak for *qDTY*$_{12.1}$. The *MtN3* class of genes is also known as the *SWEET* genes as they have been proposed to be functional in sugar transport [8], a process highly relevant to yield-under-drought. The *OsGdpD*$_{12.1}$-like proteins are also known to play an important role in cell wall formation [9]. The *OsAH*$_{12.1}$-KO line exhibited increased root growth and confirmed its negative regulation over the process.

References: 1) Faveri B et al. (2001) *KOJAK* encodes a cellulose synthase-like protein required for root hair cell morphogenesis in *Arabidopsis. Genes Dev* 15(1): 79-89. **2)** Okushima et al. (2007) ARF7 and ARF19 regulate lateral root formation via direct activation of *LBD/ASL* genes in *Arabidopsis*. *Plant Cell* 19(1): 118-130. **3)** He XJ et al. (2005) AtNAC2, a transcription factor downstream of ethylene and auxin signaling pathways, is involved in salt stress response and lateral root development. *Plant J* 44(6): 903-916. **4)** Liu et al. (2013) Functional analysis of the ABA-responsive protein family in ABA and stress signal transduction in *Arabidopsis*. *Chin Sci Bull* 58(31): 3721-3730. **5)** Sreenivasulu et al. (2012) Contrapuntal role of ABA: does it mediate stress tolerance or plant growth retardation under long-term drought stress? *Gene* 506(2):265-73. **6)** Savaldi-Goldstein et al. (2003) Alternative splicing modulation by a LAMMER kinase impinges on developmental and transcriptome expression. *Plant Cell* 15(4): 926-938. **7)** Kanneganti V and Gupta AK (2011) RNAi mediated silencing of a wall associated kinase, *Os*iWAK1 in *Oryza sativa* results in impaired root development and sterility due to anther indehiscence. *Physiol Mol Biol Plants* 17(1): 65-77. **8)** Yuan M et al. (2014) Rice *MtN3/saliva/SWEET* gene family: Evolution, expression profiling, and sugar transport. Journal of Integrative *Plant Biol Online* doi:10.1111/jipb.12173. **9)** Hayashi S et al. (2008) The glycerophosphoryl diester phosphodiesterase-like proteins SHV3 and its homologs play important roles in cell wall organization. *Plant Cell Physiol* 49(10):1522-1535.

Table S3.

NAM/NAC binding sequence and position in promoters of the 5 genes in Vandana and Way Rarem

**Gene**	**Size**		**CATGTG**		**TTNCGTR**		**TTNCGTRrc**	
	**V**	**WR**	**V**	**WR**	**V**	**WR**	**V**	**WR**
OsGdpD$_{12.1}$	2001	2002	856, 558	856, 558				
OsCesA$_{12.1}$		1932						1773
OsNod$_{12.1}$	2003	1998			616	613	999, 278	996, 275
OsGRAM$_{12.1}$	1989	2004	774	774				
OsARF$_{12.1}$	2005	2002			1789, 1717, 1655, 1039	1785,1713, 1651, 1035	1870, 1648, 925	1866, 1644, 921
OsAmi$_{12.1}$	2001	2001	1434	1434	1640	1640	1708	1708

Table S5: Statistical analysis used

$$P_{ijk} = M + R_i + B_j(R_i) + L_k + e_{ijk}$$
$$\text{Add. (\%)} = [(T_L - T_V/2)/\ T_V]*100$$

Symbol	Description
P	measurement recorded on a plot
M	mean over all plots
R	replications
B	blocks
L	lines
e	error
Additive (%)	percentage additive effect of the line
T_L	trait value for the line with the QTL
T_V	trait value of the recipient parent

Statistical Package	Purpose
CROPSTAT v 7.2 (IRRI, 2007)	Yield trials
R v. 2.8.0 (RDCT, 2008)	Physiology expts.

- IRRI: International Rice Research Institute.2007. Cropstat for Windows version 7.2.2007.2. International Rice Research Institute (IRRI). Metro Manila, Philippines.
- RDCT: R Development Core Team. 2008. A language and environment for statistical computing. R Foundation for Statistical Computing, Vienna, Austria.

Table S6: Primers used for Fine mapping

PRIMER NAME	SEQUENCE
AMI_C2_F2	TTTGCCAGCTTTGACCTTCA
AMI_C2_R2	CCATCGACCGTTGCACATTA
ARF_C3_F2	ACCTCCCGTTGCTTCTCTC
ARF_C3_R2	TCGGAGAGAATTTCGGGCTC
NAM12_PF+1656	CCACATCGGTTATGACCA
5UNAM12-R1	CGTCTCCATCGATACACCTC
GRAM_C2_F2	CACCATCTGTCCAAAGTCCA
GRAM_C2_R	GTAACTCTGCTCCGGCAACT
GPDP-C3-F1	TCGTTTATTCTATTGTTTGCCA
GPDP-C3-R1	CCATCTCCTTGGCGTGTACAA
Cesa CF1	GTGCTGTCCATATATCCTCGC
Cesa CR1	CAACCTGGACCATAGCCGCT
NOD CF2	CTACCGGATCTACAAGAGCAAG
NOD CR2	GATCTTCGTCGTGAACACC
POLe_C2_F	GCGCCAAAATTTCTTGGT
POLe_C2_R2	CTTCTCGGCGGTGATCTTGA
WAK_Pro_F	CTCTCTACTCGCCAACCACC
WAK_Pro_R	CATGAACAGCCTGGTGTCGT

Table S7: Primers used in different studies with q*DTY*$_{12.1}$

Gene Name	Locus ID	Primer	Sequence information	Purpose
OsMtN3$_{12.1}$	LOC_Os12g29220	NOD qCF3	5'-tctacgcgctgatcaagtcca-3'	RT-PCR
		NOD qCR3	5'-cgtaggcgaggtacatgacgat-3'	
OsAH$_{12.1}$	LOC_Os12g28270	AMDH_CF3	5' -ggcgtgaggcgtacatcac- 3’	RT-PCR
		AMDH_CR1	5' -ggagtactgattggtagtgtcg- 3’	
OsNAM$_{12.1}$	LOC_Os12g29330	NAM qCF3	5'-ccaagtatgtcaaggggcg-3'	RT-PCR
		NAM qCR3	5'-atgacccagtccgtcttctt-3'	
OsAFC2$_{12.1}$	LOC_Os12g27520	SER_ CF2	5' -tagtgcaaagccttccctgt- 3’	RT-PCR
		SER_ CR2	5' -cctgcggtagctttcgtaac- 3’	
OsCesA10$_{12.1}$	LOC_Os12g29300	CESA_F	5’ -gcgtcttcttcgactgcac- 3’	RT-PCR
		CESA_R	5’ -caacctggaccatagccgct- 3’	
OsEP1$_{12.1}$	LOC_Os12g29340	EXP_CF1	5' -gctgaaagcctctccatgtt- 3’	RT-PCR
		EXP_CR1	5' -gcatgatgcatagtggatgg- 3’	
OsGDP$_{12.1}$	LOC_Os12g29400	GDP_CF2	5’ -cgtggagctactacaaggtgat- 3’	RT-PCR
		GDP_CR2	5’ -catagctaacgaaccccatga- 3’	
OsWAK$_{12.1}$	LOC_Os12g29430	WAK qPCF1	5' -gcctcactactggaagaagg- 3’	RT-PCR
		WAK qPCR1	5' -tcccctctagctgatatgc- 3’	
OsPOEI19$_{12.1}$	LOC_Os12g28770	Pol Ole qCF5	5' -ctccaaccacagctacttcttgc- 3’	RT-PCR
		Pol Ole qCR5	5' -caccttctcggcggtgatcttg- 3’	
OsNAM$_{12.1}$	LOC_Os12g29330	NAM101-119F	5’ -ttgattttgccgaggtgta- 3’	Cloning
		NAM168-186RI	5’cctgctcactccaccctggaggaagcaggtcgga3’	Cloning
OsGDP$_{12.1}$	LOC_Os12g29400	ProGDP01	5' -ggcctccaaaatttatagtccca-3'	EMSA
		ProGDP02	5' -gtggagaggcctcctgtttac- 3'	
OsCesA10$_{12.1}$	LOC_Os12g29300	ProCESA01	5' -gaggcttcctgttgactggt- 3'	EMSA
		ProCESA02	5' -attgcctccgttggtgttga- 3'	
OsARF$_{12.1}$	LOC_Os12g29520	ProARF01	5' -tctgtagccccgctattctt- 3'	EMSA
		ProARF02	5' -aggtagagcggtgaggtcac- 3'	
OsMtN3$_{12.1}$	LOC_Os12g29220	ProNOD01	5' -tacccgtgcaaacaaagaacag- 3'	EMSA
		ProNOD02	5' -ggaaagtcttttggacacgc- 3'	
OsAH$_{12.1}$	LOC_Os12g28270	ProAMI01	5’ –tcgtctcgcaatttacacgt- 3’	EMSA
		ProAMI02	5’ –catggctcacgagatgtatgt- 3’	
OsGdpD$_{12.1}$ *F4*	LOC_Os12g27102	ProGdpD_F401	5’ –atctccagattcgttgcaca- 3’	EMSA
		ProGdpD_F402	5’ –atctccagattcgttgcaca- 3’	
OsGdpD$_{12.1}$ *F5*	LOC_Os12g27102	ProGdpD_F501	5’ –atctccagattcgttgcaca- 3’	EMSA
		ProGdpD_F502	5’ –atctccagattcgttgcaca- 3’	

T**able S8.** TRIM mutants

Gene	TRIM lines used	AT/KO
OsAFC2$_{12.1}$	M0032667	AT
OsAH$_{12.1}$	M0039637	KO
OsCESA$_{12.1}$	M0074686	AT
OsGDP$_{12.1}$	M0111080	AT
OsWAK$_{12.1}$	M0092628	AT
OsARF$_{12.1}$	M0093267	AT
OsPOLEI$_{12.1}$	M0066205	AT

NIL development, yield screening and statistical analysis

This study was conducted at the International Rice Research Institute (IRRI), Los Baños, Laguna, Philippines. Experiments for characterization of NILs were conducted in the dry season (DS) of 2010 to DS2012. IRRI is located at 14°13′N latitude, 121°15′E longitude, at an elevation of 21 m above mean sea level. The soil was classified as Aquandic Epiaquall, and was characterized at the physiology study sites as having a bulk density of 0.94 and 0.90 g cm^{-3} at a depth of 10-15 cm, 0.92 and 0.93 g cm^{-3} at 25-30 cm, and 0.89 and 0.91 g cm^{-3} at 45-50 cm in the upland and lowland fields, respectively, measured when the soil was dry in both treatments. Soil particles at a depth of 10-15 cm were classified as 49% clay, 34% silt, and 17% sand.

Plant material

$qDTY_{12.1}$ was identified in an $F_{3:4}$ population derived from the cross Vandana/Way Rarem (Table 1). Vandana is an upland-adapted cultivar derived from a cross between C22 and Kalakeri. This cultivar is early to mature, and low yielding but tolerant of drought, and is grown in drought-prone areas of Jharkhand and Orissa (eastern India). Way Rarem is a high-yielding, drought-susceptible upland rice cultivar from Indonesia. The yield-increasing allele in this study was derived from the susceptible parent, Way Rarem, making the tolerant parent Vandana the recipient parent for a MAB program. IR79971-B-102-B, one of the F_3-derived lines from the original population, was used as the donor for $qDTY_{12.1}$. This line was backcrossed to Vandana to develop BC_2- and BC_3-derived populations for the identification of NILs with $qDTY_{12.1}$ showing improved tolerance of drought compared with Vandana. A set of such contrasting +QTL and –QTL BC_2F_3-derived lines was used for the $qDTY_{12.1}$ physiology studies.

Generation of genotypic data

Young leaves were collected from 2-week-old plants and freeze-dried. Freeze-dried leaf samples were ground using a Geno/Grinder® (SPEX CertiPrep) and DNA was extracted by the modified CTAB method (Murray and Thomson, 1980) in deep-well plates. The quality and quantity of DNA were then checked on 0.8% agarose gel and diluted to a final concentration of 20 ng μL^{-1} with TE (Tris-EDTA) buffer. Polymerase chain reaction (PCR) was performed in 96-well polycarbonate plates according to the method described by Panaud *et al*. (1996). After the PCR was completed, 4 µL of 6× loading dye was added to each well. Four µL of the resulting solution mix was then loaded into an 8% (w/v) polyacrylamide gel (Sambrook *et al*., 1989) for size separation of the amplified DNA fragments using a mini vertical electrophoresis system (CBS Scientific, model MGV-202–33). DNA fragments were then stained with SYBR® Safe gel stain (Invitrogen) and visualized with a UV trans-illuminator.

Rice SSR markers were used for foreground, recombinant, and background selection. Markers described by Bernier *et al*. (2007) were used for selection for foreground and background. Three other markers, RM28076, RM28089, and RM28099, were also included for foreground and recombinant selection. The cM position described by Bernier *et al*. (2007) was used for the construction of chromosome maps except for the three additional markers where the cM position was calculated based on the physical distance (Mb) of these markers from RM28048. Graphical genotyping software GGT 2 (Van Berloo, 2008) was used for the construction of chromosome maps of the selected lines in various generations.

Molecular marker analysis and crossing scheme

The MAB scheme for the transfer of $qDTY_{12.1}$ into Vandana is shown in Fig. 1. $qDTY_{12.1}$ spans between RM28048 and RM28166 on chromosome 12 of the rice genome. IR79971-B-102-B, a $F_{3:4}$ line with the full segment of the QTL, was crossed twice to Vandana to develop a BC_2F_1 (241 plants). The population was screened with RM28048, RM511, and RM28166 to identify individual plants segregating for $qDTY_{12.1}$ (foreground selection). The selected plants were then screened with 42 SSR markers for the presence of the Vandana allele across the background (background selection) and two BC_2F_1 plants (IR84984-21-19 and IR84984-83-15) segregating for $qDTY_{12.1}$ and maximum background recovery were identified to develop the BC_2F_2 population. A large BC_2F_2 population (1907 plants) was developed from the identified BC_2F_1 plants and was genotyped with foreground markers RM28048, RM28130, and CG29430 (marker designed for $qDTY_{12.1}$ region) to identify lines segregating for the $qDTY_{12.1}$ locus. The 180 BC_2F_3 lines identified through this process were then saturated with six additional SSR markers, RM28076, RM28089, RM28099, RM511, RM1261, and RM28166, within the region and were screened under varying drought-stress conditions to identify high-yielding BC_2F_3-derived NILs. Lines from this population were also used for the study of QTL physiology. Six of these lines were further backcrossed to Vandana to develop a large BC3 derived population for confirmation of fine mapping results obtained in the BC2 derived population as well as to develop BC3 derived NILs. A total of 148 BC_3F_1 plants were generated and genotyped with all eight SSR markers within the QTL region. 15 BC_3F_1 plants segregating for different segments or full region of $qDTY_{12.1}$ were selected for developing a large BC_3F_2 population (2263 plants). 470 BC_3F_2 plants were identified from this population based on the segments of QTL present in them and were used for further screening for confirmation of fine mapping and identification of NILs.

BC_3F_3 lines developed from these plants were screened under drought stress and non-stress conditions. The 52 best $BC_3F_{3:4}$ lines were selected based on their field performance under stress. These lines were then evaluated under non-stress conditions for superior plant type and yield and single panicle selections were conducted. Seeds from 62 different lines coming from BC_2- and BC_3-derived populations were multiplied under non-stress conditions and confirmed for the presence of *qDTY*$_{12.1}$ along with background screening with segregating markers. Thirty-five selected lines with the *qDTY*$_{12.1}$ segment were evaluated in advanced yield trials (AYTs) under upland stress and non-stress conditions and four BC_2-derived and three BC_3-derived lines with *qDTY*$_{12.1}$, superior plant type, the highest Vandana genome recovery, and highest yield under non-stress conditions were identified. Further, these four of the seven NILs were identified and screened in repeated yield trials across season in different plot sizes to characterize the effect of QTL as well as the yield advantage of the NILs over Vandana.

Experimental conditions

Drought screening in yield trials to develop NILs

All populations were screened in upland conditions under severe stress, moderate stress, and non-stress treatments. The $BC_2F_{3:4}$ and $BC_3F_{3:4}$ lines with *qDTY*$_{12.1}$ developed through MAB were screened using an α-lattice design along with Vandana, Way Rarem, in two replications of 2-m and 1.5-m single-row plots for severe stress and 2-m and 1-m single-row plots for non-stress conditions. A 0.25-m row-to-row spacing was followed in all the experiments. The NILs identified from these trials were screened in advanced yield trials (AYTs) using an α-lattice along with the two parents and popular upland checks across different seasons and stress severities. These yield trials had three to four replications of 2-m, four to eight row plots. Seeds

were dry-direct-seeded in aerobic soil using a seeding rate of 2.5 g per linear meter of row. Fertilizer and crop management practices were followed as described by Venuprasad *et al*. (2009). In all stress experiments, trials were sprinkler-irrigated twice a week during establishment and early vegetative growth. At 35 days after seeding, stress was initiated by withholding irrigation and plots were irrigated only when the soil water tension fell below –50 kPa at 30-cm soil depth. At this soil water potential, most lines wilted and exhibited leaf drying. This type of cyclic stress is considered to be efficient in screening for drought tolerance in populations consisting of genotypes with a broad range of growth duration (Lafitte *et al*., 2004) and ensures that all lines receive adequate stress during reproductive development. Upland non-stress trials received the same cultural practices as the stress trials except that irrigation was continued twice a week up to 10 days before harvest. The trials were irrigated to field capacity at each irrigation and no flooding was allowed.

Observations recorded

In all yield trials, days to 50% flowering (DTF), mean plant height at maturity (PH), grain yield, and biomass were recorded. DTF was recorded as the number of days from sowing until 50% of the plants in a plot had flowering tillers. Plant height of three plants from each plot was measured at maturity from ground level to the tip of the tallest tiller and averaged to get the PH. Grain yield from each plot was harvested at physiological maturity, dried to a moisture content of about 14%, and weighed (Venuprasad *et al*., 2009). Biomass was sampled by selecting a uniform section 50 cm in length in each plot and harvesting the plants in this section at ground level. Biomass samples were then oven-dried, weighed, and threshed. Harvest index was estimated as the ratio of grain weight to whole-plant weight of the biomass sample. Grain measurements were

conducted on non-stress grains harvested from the NILs, parents and checks. Length and width of grains were calculated from observations taken from 10 grains of each line. L:W ratio was calculated based on the mean length and width data.

Statistical analysis

Data of all experiments for computation of means and standard error of difference (SED) were analyzed using CROPSTAT version 7.2.3 (http://archive.irri.org/science/software/cropstat.asp). Mixed model analysis of data was carried out using the model

$$y_{ijk} = \mu + g_i + r_j + b_{lj} + e_{ijk}$$

where μ is the overall mean, g_i is the effect of the ith genotype, r_j is the effect of the jth replicate, b_{lj} is the effect of the lth block within the jth replicate and e_{ijk} is the error. Combined analysis was conducted for the lowland experiments to obtain line means across years under stress and non-stress conditions. Genotypic effects were considered fixed and the replicates and block effects were random. Broad-sense heritability (*H*) of the traits for single years was calculated as shown below:

Additive effect of the line with the QTL was computed as

$$A = \left[\frac{(T'/T)}{T}\right] \times 100$$

where A is the percentage additive effect of the line over the recipient parent (Vandana), *T'* is the trait value for the line, and *T* is the trait value of the recipient parent (Vandana).
